# Mineral Preservatives in the Wood of Stradivari and Guarneri

**DOI:** 10.1371/journal.pone.0004245

**Published:** 2009-01-22

**Authors:** Joseph Nagyvary, Renald N. Guillemette, Clifford H. Spiegelman

**Affiliations:** 1 Department of Biochemistry and Biophysics, Texas A&M University, College Station, Texas, United States of America; 2 Department of Geology and Geophysics, Texas A&M University, College Station, Texas, United States of America; 3 Department of Statistics, Texas A&M University, College Station, Texas, United States of America; Western Illinois University, United States of America

## Abstract

Following the futile efforts of generations to reach the high standard of excellence achieved by the luthiers in Cremona, Italy, by variations of design and plate tuning, current interest is being focused on differences in material properties. The long-standing question whether the wood of Stradivari and Guarneri were treated with wood preservative materials could be answered only by the examination of wood specimens from the precious antique instruments. In a recent communication (Nature, 2006), we reported about the degradation of the wood polymers in instruments of Stradivari and Guarneri, which could be explained only by chemical manipulations, possibly by preservatives. The aim of the current work was to identify the minerals from the small samples of the maple wood which were available to us from the antique instruments. The ashes of wood from one violin and one cello by Stradivari, two violins by Guarneri, one viola by H. Jay, one violin by Gand-Bernardel were analyzed and compared with a variety of commercial tone woods. The methods of analysis were the following: back-scattered electron imaging, X-ray fluorescence maps for individual elements, wave-length dispersive spectroscopy, energy dispersive X-ray spectroscopy and quantitative microprobe analysis. All four Cremonese instruments showed the unmistakable signs of chemical treatments in the form of chemicals which are not present in natural woods, such as BaSO_4_, CaF_2_, borate, and ZrSiO_4_. In addition to these, there were also changes in the common wood minerals. Statistical evaluation of 12 minerals by discriminant analysis revealed: a. a difference among all four Cremona instruments, b. the difference of the Cremonese instruments from the French and English antiques, and c. only the Cremonese instruments differed from all commercial woods. These findings may provide the answer why all attempts to recreate the Stradivarius from natural wood have failed. There are many obvious implications with regard to how the green tone wood should be treated, which chould lead to changes in the practice of violin-making. This research should inspire others to analyze more antique violins for their chemical contents.

## Introduction

For centuries, violin-makers have tried in vain to match the high standards of excellence set in the first half of the 18th century by the two legendary masters of the craft, Antonio Stradivari and Joseph Guarneri del Gesù in Cremona, Italy. Since craftsmen of our age have employed all the traditional know-how of the art and have also been aided increasingly by volumes of acoustical research, their failure is hard to explain. The mechanical properties of naturally seasoned spruce and maple were thoroughly studied by several investigators [1], [2], [3] who concluded that high stiffness and low density should be the criteria in selecting the best wood. Yet the results with the best commercial wood remained unconvincing. It is possible that, due to the orthotropic nature of the wood [1], [3], the given ratio of its mechanical constants is not what it should ideally be for the best acoustical outcome. Such considerations would justify the suggestion that the trees grown during the Maunder Minimum could have had different and better mechanical properties [4].

The proposition that the answer may lie in the material differences caused by an ancient and forgotten practice of wood preservation has surfaced many times but it received less than due attention. The 1987 exhibit of documents from the State Archives of the Serenissima Republic of Venice [5] provided evidence that the wood supply was delivered through the water-ways and could have been treated with chemical preservatives for the use by all sorts of trades. The beneficial effect of salts on the wood of musical instruments was first noted by the French author Palissy in 1580 according to the historical research of R. Gug [6].

The wood of the great masters received only little attention by scientists in the past, and the focus was on the area penetrated by the ground layer of the finishing materials [7], [8], [9]. The varnish and the wood of a Stradivarius cello were subjected to analysis by ion backscattering [10], but the inaccuracies of the method did not allow conclusions beyond showing the presence of several trace elements. The first indication that the entire instrument wood of the famous Cremona masters could have received some kind of special aqueous treatment was advanced by Nagyvary [11] in the form of scanning electron microscopic (SEM) images of internal spruce sapwood samples. The micrographs from the violins of Stradivari, Guarneri and Guadagnini revealed the remnants of microorganisms, and the Guarneri also showed mineral deposits. In contrast, Barlow and Woodhouse found nothing remarkable in their SEM study of the morphology of spruce samples from Italian musical instruments [12]. They concluded that the wood of the masters had not been submerged in water for a prolonged period of time, as done in the present process of “ponding” of logs. However, SEM by itself is not a suitable method to prove the point and exclude the possibility of past aqueous treatments with chemicals, including boiling for short periods of time, which could cause important chemical and morphological changes on a smaller scale. The conclusion of the British workers was not backed up by mineral analysis, which would have been the simplest way to prove the absence of aqueous treatment.

More recently, the powerful methods of solid-state NMR and FTIR spectroscopy were employed by Nagyvary *et al.* [13] for the study of maple wood from the backs of instruments made by Stradivari and Guarneri del Gesù in comparison with the maple from antique French and English instruments. The Italian samples revealed massive chemical changes in the wood polymers while the others differed only slightly from the recent natural wood. The observed changes went well beyond what one could see after boiling in water. Thus, there could be no doubt that these particular woods were chemically treated, and the only question concerns the specifics of the chemical treatment.

In the present study we report on the mineral analysis of the ashes from the previously used maple samples [13], to which we could also add an additional sample from an early Guarneri del Gesù violin. Some years ago, the mineral composition of various trees was determined by classical methods requiring relatively large quantities of wood [14]. We have employed the methods of backscattered electron (BSE) imaging, X-ray qualitative analysis by energy dispersive spectroscopy (EDS), X-ray quantitative analysis by wavelength dispersive spectroscopy (WDS), and WDS X-ray element distribution imaging. The results suggest that the wood used by Stradivari and Guarneri was indeed treated by mineral preservatives. Such a process could have caused extensive chemical changes in the organic matrix and, therefore, could have impacted the acoustical properties of the wood. These findings have far-reaching consequences for the art of violin-making if the goal is indeed the reproduction of the great Cremonese standards.

## Materials and Methods

### Materials

Wood samples from the instruments of Stradivari and Guarneri del Gesù were taken from the interior of the backs during the repair of cracks when the restorer removed thin shavings of old wood several millimeter deep from an area approx. 6 to 8 cm^2^; the outer discolored layers were excluded. The samples were derived from the maple backs of the following provenance: (a) a violin by Stradivari, 1717 CE, (b) a cello of Stradivari, 1731 CE, (c) a violin by Guarneri del Gesù, 1741 CE, (d) a violin by Guarneri del Gesù from the mid-1730s, (e) a violin by Gand-Bernardel of Paris, mid-1840s, (f) a viola by Henry Jay from London, 1769 CE. The last two instruments were salvaged from a house fire and were exposed to a brief period of heat. For comparisons, we used maple tone wood from all important European regions such as Bosnia, Slovenia, Slovakia, Germany and also from southwest China. Such samples were either purchased from commercial companies such as International Violin Co., Baltimore, MD; David Morse Violins, Soquel, CA; Rivolta Co., Desio, Italy; ViolinMaple Co., Bratislava, Slovakia, or they were donated by private individuals who acquired them on locations. Although we asked for naturally seasoned wood, some contamination could not be excluded since the practice in some locations includes spraying of the logs with fungicides. Unusual Na values found in one Bosnian and one Slovenian samples could be due to such contamination.

#### Sample preparation

Ash samples were prepared from the wood shavings using the following technique. Between 20 and 80 mg of wood shavings were weighed and transferred to Coors porcelain covered crucibles; only 13 mg of Cremona soot was used because of the smaller expected loss of volatiles. The crucibles were inserted into a cold Lindberg muffle furnace and the furnace was set to 500°C and turned on. After 2 hours, crucibles were removed from the furnace and allowed to cool. Ash was removed from the crucibles and pressed between 2 highly-polished (0.25 µm surface finish) stainless steel cylinders to form a 1–3 mm diameter pellet. Each pellet was transferred onto the surface of conductive carbon tape and coated with 15 nm of spectroscopically pure amorphous carbon in a Ladd carbon evaporator to make the pellet conductive under an electron beam.

### Analytical Methods

Analyses were carried out on a Cameca sx50 electron microprobe equipped with four wavelength dispersive X-ray spectrometers (WDS) and an energy dispersive X-ray spectrometer (EDS) using standard microprobe imaging and microanalytical methods [15], [16]. The WDS spectrometers are capable of detecting and qualitatively and quantitatively analyzing elements from Z = 4 (Be) through Z = 92 (U). The Li-drifted Si detector with ultra-thin window in the EDS system is capable of detecting and qualitatively analyzing elements from Z = 6 (C) through Z = 92 (U). Electron imaging was performed using a six component backscattered electron (BSE) detector operated in atomic number mode, where the brightness in an image is proportional to the mean atomic number of the area being imaged. X-ray elemental distribution images (“maps”) were acquired by moving the stage containing the sample in 2 µm steps in a 512 by 512 grid beneath the fixed 1 µm diameter beam; dwell time at each step (pixel) was 15 ms. Each of the four WDS spectrometers was set to the major X-ray emission line of an element, with an additional pass required for each additional set of four elements. The brightness of each pixel in the image is proportional to the number of X-rays from that element detected at that point on the sample.

EDS qualitative analyses in the form of X-ray spectra were acquired on the pressed powder pellets. The sizes of the areas analyzed varied from about 1 µm up to 1 mm. The smaller sizes were used to examine different BSE heterogeneous areas, while the larger sizes were used to average as large an area as possible on the pellets. EDS quantitative analyses were not attempted because of the larger errors associated with EDS analyses compared to WDS analyses.

WDS quantitative analyses were performed on the samples using standard methods after careful calibration using well characterized Smithsonian [17], [18] and C. M. Taylor Inc. [19] reference standards. Calibration for all elements was performed at the beginning of each analytical session, followed by the analysis of known compounds as standard checks. WDS analyses were carried out on 20 µm diameter spots distributed over the surface of the pressed powder pellets in as representative a manner as possible; 20 µm is the largest area that can be analyzed in a single WDS analysis as determined by the constraints of spectrometer geometry. A larger number of points was selected on the more heterogeneous samples (as determined by BSE imaging) in order to obtain better averages for those samples.

Boron was detected semi-quantitatively using WDS spectral scans centered on the boron K alpha X-ray emission line and carried out under identical conditions on the pressed powder pellets.

### Statistical analysis

Only elements that were measured for all instruments and woods in Tables 1 and 2 were used in the multivariate statistical analysis. These 12 elements (cations as oxides) were Cl, Na_2_O, K_2_O, CaO, MgO, SiO_2,_ Al_2_O_3_, P_2_O_5_, SO_3_, FeO, MnO, TiO_2_. The full set of data, compiled in Supplemental Information (Table S1), included 95×12 values for the Stradivarius violin, 75×12 for the early Guarneri and 30×12 for the rest, with the exception of the German maple which had only 15×12 data points. The differences between the wood samples could be best seen in plots based on canonical axes designed to separate the instruments as much as possible. These axes are linear combinations of the elements that were measured in all the instruments and commercial woods under study. The statistical technique used to construct the axes is known as discriminant analysis; see Johnson and Wichern [20]. All calculations were done using JMP [21] statistical software and the quadratic discriminant analysis module. We used these axes only for plotting. It was not our purpose to create an assignment rule for new measurements to one of these instrument types as is typical with discriminant analysis.

**Table 1:** Quantitative analyses of musical instrument ashes.

Norm Anal	F	Cl	Na_2_O	K_2_O	CaO	MgO	SiO_2_	Al_2_O_3_	P_2_O_5_	SO_3_	BaO	FeO	MnO	CuO	ZnO	TiO_2_
LLD	0.27	0.04	0.10	0.05	0.08	0.07	0.10	0.06	0.10	0.11	0.18	0.15	0.15	0.28	0.35	0.22
StradCello(15)		1.26	6.11	15.93	40.17	10.15	4.62	1.98	4.76	13.54		1.32	0.16			0.21
*Strad Cello SD*		*0.50*	*2.00*	*7.06*	*9.46*	*4.00*	*3.40*	*2.05*	*1.79*	*3.24*		*1.90*	*0.08*			*0.34*
Strad Vln(95)	0.84	11.34	23.26	25.26	15.45	4.48	3.89	0.50	1.59	9.70	1.26	0.81	0.11	1.11	0.32	0.08
*Strad Violin SD*	*0.63*	*6.59*	*12.14*	*8.57*	*6.51*	*3.11*	*9.51*	*0.61*	*0.87*	*4.17*	*5.33*	*0.87*	*0.11*	*2.47*	*0.56*	*0.12*
Guarneri(80)	0.08	0.19	7.61	15.70	24.63	3.27	6.81	5.29	1.89	23.11	0.14	8.06	0.17	0.14	0.34	0.55
*Guarneri SD*	*0.06*	*0.06*	*2.02*	*2.72*	*5.86*	*1.27*	*3.99*	*3.12*	*0.72*	*4.01*	*0.12*	*5.25*	*0.12*	*0.08*	*0.23*	*0.48*
HenryJay(29)	0.22	0.35	4.63	34.18	37.66	8.11	2.05	0.62	6.71	4.26	0.31	0.27	0.24	0.18	0.16	0.05
*HenryJay SD*	*0.12*	*0.09*	*2.22*	*3.89*	*3.74*	*1.07*	*2.50*	*1.12*	*1.77*	*0.83*	*0.11*	*0.33*	*0.06*	*0.13*	*0.17*	*0.08*
Gand-Bern(30)	0.18	0.24	5.18	19.95	50.89	10.66	0.81	0.14	5.00	5.06	0.18	0.98	0.31	0.18	0.18	0.06
*Gand-Bern SD*	*0.09*	*0.04*	*1.42*	*4.38*	*5.46*	*1.55*	*1.51*	*0.26*	*0.89*	*1.37*	*0.14*	*4.13*	*0.09*	*0.09*	*0.13*	*0.10*
Soot (30)	0.26	1.07	0.70	9.79	40.17	5.76	5.60	1.94	4.76	27.79	0.08	0.81	0.07	0.13	0.96	0.12
*Soot SD*	*0.10*	*0.36*	*0.17*	*1.68*	*4.41*	*1.74*	*3.56*	*1.21*	*0.84*	*4.15*	*0.05*	*0.36*	*0.04*	*0.08*	*0.21*	*0.18*

Analyses were carried out on a Cameca sx50 electron microprobe equipped with four WDS spectrometers. All analyses were performed with a 15 kV, 10 nA, 20 µm diameter beam; standardization and standard checks using well-characterized reference materials were done before each analytical session. The numbers represent average wt% values, normalized to 100%; number of sites analyzed is in parentheses. SD: standard deviation. It is customary to present the values as oxides. The lower limit of detection (LLD) for each element or oxide is given at the top of the table. Abbreviations: Guarneri designates the ash of the early Guarneri violin, Gand-Bern that of the Gand-Bernardel violin.

**Table 2:** Quantitative analyses of various commercial maple ashes.

Norm Anal	F	Cl	Na_2_O	K_2_O	CaO	MgO	SiO_2_	Al_2_O_3_	P_2_O_5_	SO_3_	BaO	FeO	MnO	CuO	ZnO	TiO_2_
LLD	0.27	0.04	0.10	0.05	0.08	0.07	0.10	0.06	0.10	0.11	0.18	0.15	0.15	0.28	0.35	0.22
Grmn(15)		0.86	3.67	46.61	32.37	7.34	1.27	0.50	2.44	1.24		0.03	3.68			0.04
*Grmn SD*		*0.36*	*2.86*	*6.70*	*6.47*	*1.50*	*0.85*	*0.25*	*1.54*	*0.63*		*0.06*	*0.70*			*0.07*
Slvk_ht(30)	0.34	2.23	3.74	15.71	52.79	15.17	1.06	0.14	3.76	3.87	0.04	0.54	0.23	0.16	0.15	0.07
*Slvk_ht SD*	*0.20*	*1.18*	*3.18*	*7.84*	*8.08*	*3.44*	*2.23*	*0.44*	*0.90*	*0.96*	*0.04*	*1.07*	*0.10*	*0.11*	*0.14*	*0.07*
Slvk_sp(30)	0.35	0.65	1.80	40.46	31.36	9.92	1.70	0.21	9.01	3.54	0.05	0.52	0.11	0.15	0.10	0.07
*Slvk_sp SD*	*0.25*	*0.33*	*1.24*	*6.71*	*4.75*	*1.74*	*2.67*	*0.45*	*2.61*	*0.84*	*0.05*	*1.21*	*0.06*	*0.14*	*0.11*	*0.07*
Slvk_95(30)	0.50	0.78	3.23	33.02	34.50	14.14	1.05	0.07	7.72	2.28	2.02	0.14	0.20	0.19	0.12	0.03
*Slvk_95 SD*	*0.18*	*0.37*	*2.24*	*4.42*	*3.98*	*3.19*	*1.08*	*0.18*	*1.51*	*0.43*	*2.69*	*0.32*	*0.07*	*0.11*	*0.10*	*0.05*
Slvn1_ht(30)	0.32	0.93	3.66	26.45	44.46	7.82	5.31	1.69	4.29	3.67	0.27	0.54	0.22	0.14	0.14	0.10
*Slvn1_ht SD*	*0.19*	*0.59*	*2.53*	*8.70*	*7.23*	*1.65*	*5.50*	*2.03*	*1.15*	*0.90*	*0.09*	*0.56*	*0.08*	*0.09*	*0.14*	*0.12*
Slvn1_sp(30)	0.25	2.93	11.72	14.09	44.55	9.52	1.54	0.30	4.90	7.62	0.22	0.94	0.25	0.20	0.92	0.04
*Slvn1_sp SD*	*0.15*	*1.63*	*4.01*	*3.88*	*9.74*	*2.47*	*1.71*	*0.60*	*1.58*	*2.44*	*0.09*	*1.24*	*0.08*	*0.12*	*0.43*	*0.06*
Slvn2_ht(30)	0.21	0.36	3.50	32.00	42.26	7.34	4.33	1.30	3.82	3.38	0.51	0.35	0.20	0.16	0.12	0.13
*Slvn2_ht SD*	*0.09*	*0.13*	*1.70*	*5.28*	*3.97*	*1.45*	*4.55*	*1.70*	*1.04*	*0.58*	*1.34*	*0.42*	*0.06*	*0.07*	*0.11*	*0.23*
Slvn2_sp(30)	0.19	1.37	4.86	30.05	38.78	8.13	1.78	0.18	8.59	4.86	0.32	0.13	0.26	0.19	0.24	0.04
*Slvn2_sp SD*	*0.11*	*2.40*	*2.69*	*8.20*	*6.40*	*1.57*	*1.67*	*0.45*	*2.44*	*0.94*	*0.10*	*0.17*	*0.06*	*0.10*	*0.19*	*0.07*
Chns(30)	0.52	0.27	2.58	16.66	54.91	12.29	1.44	0.21	4.21	4.69	0.34	0.35	1.11	0.25	0.10	0.07
*Chns SD*	*0.28*	*0.06*	*1.15*	*4.53*	*3.90*	*1.06*	*2.20*	*0.26*	*0.63*	*1.46*	*0.14*	*0.51*	*0.21*	*0.13*	*0.15*	*0.07*
Bosn1(30)	0.08	0.11	12.55	16.11	31.24	28.59	0.14	0.02	4.98	3.56	0.28	0.07	1.93	0.14	0.18	0.02
*Bosn1 SD*	*0.06*	*0.05*	*1.17*	*1.99*	*1.37*	*1.88*	*0.15*	*0.03*	*0.87*	*0.67*	*0.08*	*0.11*	*0.40*	*0.10*	*0.10*	*0.03*
Bosn2(30)	0.08	0.18	1.10	27.41	33.38	30.10	0.17	0.03	4.33	2.14	0.22	0.04	0.49	0.16	0.15	0.02
*Bosn2 SD*	*0.08*	*0.16*	*0.24*	*4.08*	*2.47*	*1.95*	*0.15*	*0.09*	*1.17*	*0.38*	*0.09*	*0.05*	*0.09*	*0.10*	*0.13*	*0.02*
Bosn3(30)	0.13	0.16	1.58	15.95	47.65	26.45	0.36	0.03	4.13	2.38	0.21	0.05	0.58	0.13	0.15	0.04
*Bosn3 SD*	*0.09*	*0.03*	*1.23*	*4.87*	*4.35*	*3.50*	*0.18*	*0.04*	*1.21*	*0.35*	*0.08*	*0.06*	*0.07*	*0.11*	*0.11*	*0.05*
Bosn4 30)	0.06	0.19	4.35	8.90	46.45	30.87	0.44	0.01	4.21	3.96	0.05	0.07	0.12	0.16	0.14	0.02
*Bosn4 SD*	*0.06*	*0.03*	*1.12*	*2.38*	*1.68*	*1.93*	*0.26*	*0.02*	*0.78*	*0.35*	*0.04*	*0.06*	*0.06*	*0.10*	*0.10*	*0.03*
Bosn5(30)	0.04	0.17	1.53	20.99	35.43	33.99	0.18	0.02	4.42	2.17	0.17	0.04	0.49	0.18	0.16	0.03
*Bosn5 SD*	*0.04*	*0.04*	*0.73*	*5.68*	*6.16*	*5.06*	*0.21*	*0.02*	*1.23*	*0.52*	*0.07*	*0.05*	*0.10*	*0.09*	*0.12*	*0.04*
Bosn(30)	0.09	0.32	1.25	22.59	39.15	27.87	0.21	0.02	4.90	2.41	0.27	0.04	0.60	0.16	0.10	0.03
*Bosn6 SD*	*0.05*	*0.04*	*0.46*	*5.85*	*2.87*	*3.11*	*0.17*	*0.03*	*1.30*	*0.49*	*0.07*	*0.04*	*0.09*	*0.09*	*0.10*	*0.04*

Analyses were carried out on a Cameca sx50 electron microprobe equipped with four WDS spectrometers. All analyses were performed with a 15 kV, 10 nA, 20 µm diameter beam; standardization and standard checks using well-characterized reference materials were done before each analytical session. The numbers represent average wt% values, normalized to 100%; number of sites analyzed is in parentheses. SD: standard deviation. The lower limit of detection (LLD) for each element or oxide is given at the top of the table. Abbreviations of wood ashes. Grmn: German; Slvk_ht: Slovak heartwood; sp: sapwood; Slvk_95: Slovakian wood from 1995; Slvn_1 and Slvn_2 are samples from the same Slovenian maple board but from different depth; Chns: Chinese; Bosn1–Bosn6 designate samples from 6 different Bosnian trees.

## Results

### Backscattered electron (BSE) imaging

**Figure 1:**
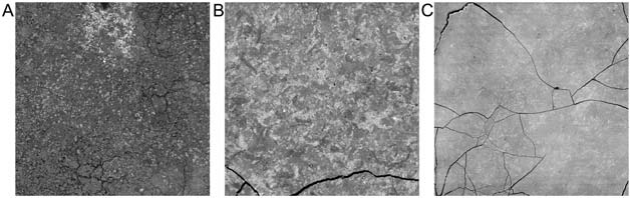
Backscattered electron images. BSE images of 1 mm^2^ areas of the pressed powder pellets made from the ash of the (A) Stradivari violin, (B) early Guarneri violin, and (C) one of the commercial Bosnian maples. The two violin ash samples exhibit a higher degree of the BSE brightness variability and hence compositional heterogeneity than the Bosnian maple.

The controlled burning of the wood shavings yielded approximately 0.5 to 1.0 mg quantities of ashes, which were pressed into pellets as described above. The first preliminary assessment of the possible differences was made by backscattered electron (BSE) imaging targeting an area of 1 mm^2^. These are shown in Fig. 1 for the Stradivari violin (A), the early Guarneri violin (B) and one of the 12 recent commercial wood samples (C). On these gray-scale images the degree of contrast correlates with chemical heterogeneity, and the brightness is a function of the mean atomic number of the elements. By these criteria, one can see significant differences among these images.

### Elemental distribution images

**Figure 2:**
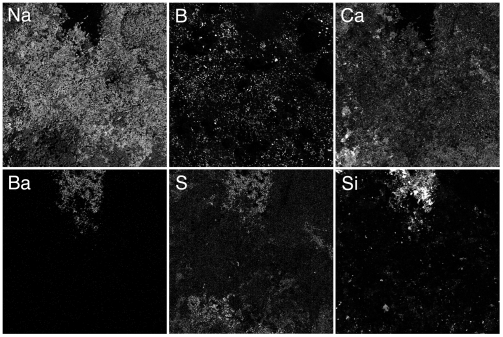
Elemental distribution images in the Stradivari violin ash. WDS-based imaging of 1 mm^2^ areas was employed for the elements Na, B, Ca, Ba, S, and Si. Note the higher degree of variability in the distribution of the elements B, Ba, S, and Si. Partial overlapping images suggest the presence of BaSO_4_, CaSO_4_ and Na-borate.

The use of WDS detectors allowed for the imaging of individual elements. We acquired such X-ray maps covering an area of 1 mm^2^ for the anticipated elements Al, B, Ba, Ca, Cl, Cu, F, Fe, K, Mg, Na, P, S, Si and Zn. These images were useful to show the uneven distribution of elements within the ash pellet, and they also helped in identifying some chemical compounds that were originally applied to the wood, such as CaSO_4_, BaSO_4_, Na-borate and CaF_2_. In general, the distribution of the major elements Ca, K, and Na is more even than those of the minor elements. Fig. 2 shows some representative images of elements from the ash of the Stradivari violin wood. Noteworthy is the presence of boron, an element normally not found in wood, which can be easily overlooked because of its low energy X-ray emission line which must be detected using a WDS spectrometer equipped with a special large d-spacing pseudocrystal. The comparison of its map with that of Na suggests that it was probably applied as Na-borate (borax). The presence of BaSO_4_ is also indicated by the coincidence of the Ba and S images. It is not a constituent of wood, and its presence must be due to a deliberate chemical manipulation.

**Figure 3:**
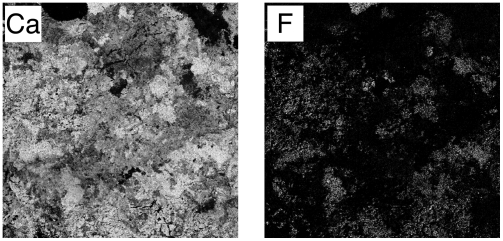
Elemental distribution images in the late Guarneri violin. WDS-based imaging of 1 mm^2^ areas was employed for the elements Ca and F. Note that all F-bearing regions also contain Ca. Thus the particles constitute CaF_2_.

When a similar WDS-based imaging was applied to the late period Guarneri violin ash, it led to the discovery of CaF_2_, a compound otherwise not found in wood (Fig. 3). It is interesting to note that none of the other violins had any significant amounts of fluoride, as it could be determined by WDS X-ray distribution maps.

**Figure 4:**
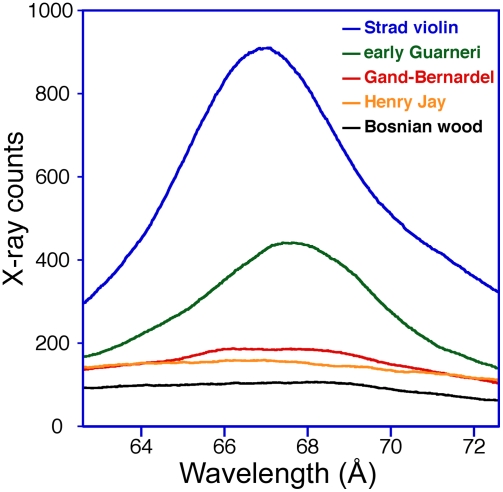
The detection of boron. WDS spectral scans over the boron peak are shown on the ash pellets of the Stradivari violin (blue), early Guarneri violin (green), Gand-Bernardel violin (red), Henry Jay viola (orange) and commercial Bosnian maple (black).

Particular care was taken to search for borate in all samples because of its role as an insecticide-fungicide of long-standing usage. Boron was found in both instruments of Stradivari and the early period Guarneri; it was absent in the late Guarneri and in all the commercial maple samples. The amount of borate in the Gand-Bernardel and perhaps in the Henry Jay was slightly beyond its natural abundance in wood. This is apparent in Fig. 4 which shows WDS spectral scans over the boron peak on the Stradivari violin, early Guarneri violin, Gand-Bernardel violin, Henry Jay viola, and recent commercial Bosnian maple ash pellets.

### EDS Spectra

**Figure 5:**
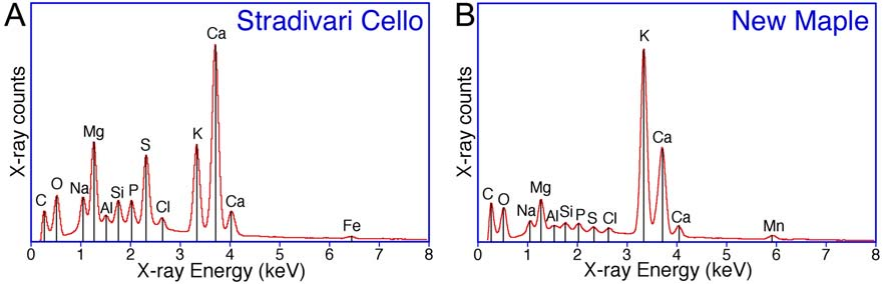
EDS spectra of ash from the Stradivari cello and from new maple. A: ash from Stradivari cello, B: recent maple. Each spectrum represents the average of ten spectra acquired at different >100 µm diameter areas on each pellet over a total acquisition time of 1200 seconds.

**Figure 6:**
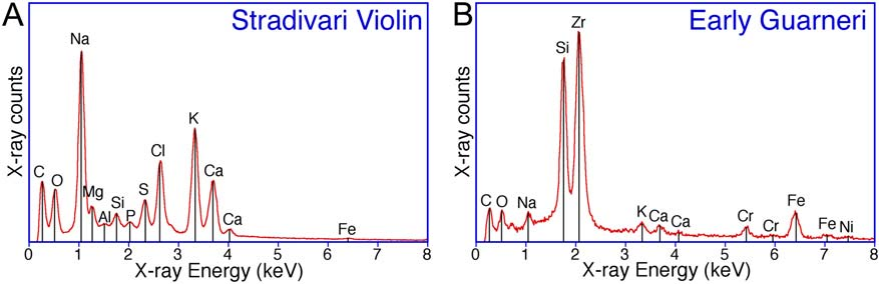
EDS spectra of ash from violins of Stradivari and Guarneri. The Stradivari violin spectrum A was acquired over a large representative 400 µm diameter area of the pellet, while the Guarneri violin spectrum B represents a small BSE bright Zr rich area.

The EDS method was useful in identifying elements of mineral clusters in small areas, and thus establishing the degree of heterogeneity of the ashes. At the largest setting of 1 mm^2^ and repeated sampling, the average spectrum has become a reasonable semi-quantitative representation of the overall elemental composition. The EDS spectrum of Figure 5A represents the average of 10 spectra taken on the ash of the Stradivari cello, which is noticeably different from the average spectrum of recent maple of Fig. 5B, albeit not so obviously different as the 3 violins of Cremona. The spectra of the Stradivari violin taken at several locations show the presence of NaCl well above its natural occurrence in wood (Fig. 6A). The survey of the ash pellets of both Guarneri violins revealed a trove of unusual components. A particular site of the early period Guarneri contains crystals of ZrSiO_4_ besides Fe and Cr salts (Fig. 6B). The spectra of the Gand-Bernardel and Henry Jay instruments (not shown here) revealed only minor deviations from those of the 11 commercial samples tested in this study.

### Quantitative measurements

Electron microprobe WDS analysis was employed for the quantitative determination of the elements from all the above samples, with one exception. Due to a temperature control problem, the late Guarneri sample was overheated to almost 700°C and lost most of its K; this sample was not used in this analysis. Because of the volatility of K [22], we had to keep the combustion temperature under 550°C. We also analyzed the composition of chimney soot from Cremona. The results of the elemental analysis of the musical instruments ash are compiled in Table 1, those of the commercial woods in Table 2, where the numbers represent wt% elements or oxides normalized to 100% total. The number of 20 µm diameter circular areas that were analyzed and averaged was 95 for the Stradivarius violin, 80 for the early Guarneri and 30 for the rest. The noticeable feature of this data set is the high standard deviation for several elements which can be due to several factors, such as the uneven distribution of the natural minerals in the wood, and the uneven uptake of extraneous minerals. Particles of the insoluble BaSO_4_ and silica would be capable of deep penetration only within the vessels of the wood, and this explains the rare occurrence of such particles within the ash. The heterogeneity of the ash, especially that from the musical instruments, can clearly be seen in X-ray maps (Fig. 2, Fig. 3) and in BSE images (Fig. 1).

The composition of the Stradivari violin ash differs significantly from those of the commercial wood samples in several regards. Its Na_2_O content is 11 times that of the Bosnian average, 5 times that of the Slovenian and 8 times that of the Slovakian maple. The respective ratios for Cl are 52, 11 and 7. Both of these sets of comparisons are supported by the relevant unequal variance t-test for equality of means and all have p-values less than 0.0001. In addition the Strad violin has a much larger variance than the other wood samples for Na_2_O and Cl, p<0.0001. It also contains more SiO_2_, SO_3_, BaO, FeO and CuO but less of CaO, MgO and phosphate. It exhibits an unnatural K/Na ratio. Borate data were not included in this table.

The Stradivarius cello ash was analyzed ten years ago, and at that time we did not include the elements Ba, F, Cu and Zn. The main differences to natural wood are apparent in the values for SO_3_, SiO_2_, and FeO. Unlike the Stradivari violin, the cello ash retained its phosphate. These findings suggest that the cello wood was treated differently from the violin wood, and this might have had something to do with the practical aspect of soaking a large piece of wood.

Of the two Guarneri violins, only the early period Guarneri was subjected to quantitative microprobe analysis. Unlike the later Guarneri, this one had very little fluoride and chloride in its ash. This makes it unlikely that it had ever been in sea water. It had more Na_2_O, SiO_2_, Al_2_O_3_, SO_3_, FeO and ZnO than any of the commercial woods. It also contained boron, chromium and zirconium, which were not included in Table 1. The early Guarneri violin also differs from the Stradivari violin, notably with regard to the respective Na_2_O, Cl, Al_2_O_3_, and FeO contents. The wood minerals of the instruments of H. Jay and Gand-Bernardel are significantly different from the Cremona instruments; otherwise the values fall within the range of natural woods.

In a few of our maple samples from commercial stocks, we analyzed both the sapwood and the heartwood, whenever we could identify them, since their values are often different. We assume, but cannot be fully certain, that our antique samples derived from sapwood or a transitional area between sapwood and heartwood. Since tradition had it that the maple of Stradivari was of Bosnian origin, we analyzed 6 different Bosnian maple samples. The most distinctive feature of their elemental composition is the high values of Mg, which must be due to the dolomite minerals of the local soil. The only way to reconcile the Ca/Mg ratios of the Stradivari and Guarneri wood with a Bosnian origin would be to assume that the excess Mg-salts had been extracted. Slovenian maple would have been a more likely source for Stradivari and Guarneri on the basis of the respective Ca/Mg ratios. Although our intention was to include only natural and unadulterated wood in our analyses, it appears that our Bosnian sample no. 1 and the Slovenian wood could have been treated by salt. Their ash revealed the presence of NaCl well beyond those seen in the other samples. We also included Chinese and Slovakian maple woods in our study since they are commonly available these days.

Finally, we analyzed chimney soot from Cremona by several types of analysis including the microprobe, and its composition was included in Table 2. According to a historical source [23], alcoholic extract of soot was liberally applied to the wood of violins. The ash from the soot reflected the very fine particle size of the original fly-ash. Unfortunately, our 30-year old soot sample was probably deposited from a combination of wood and coal burning, and it may not be representative of soot produced in the 18^th^ century. The high value of SO_3_ could be best explained by assuming low-grade coal as a major source. Even with this caveat, the similarities of the soot and violin values are intriguing and give some credence for the possible use of soot in the processing of the old violin wood.

### Statistical analysis

**Figure 7:**
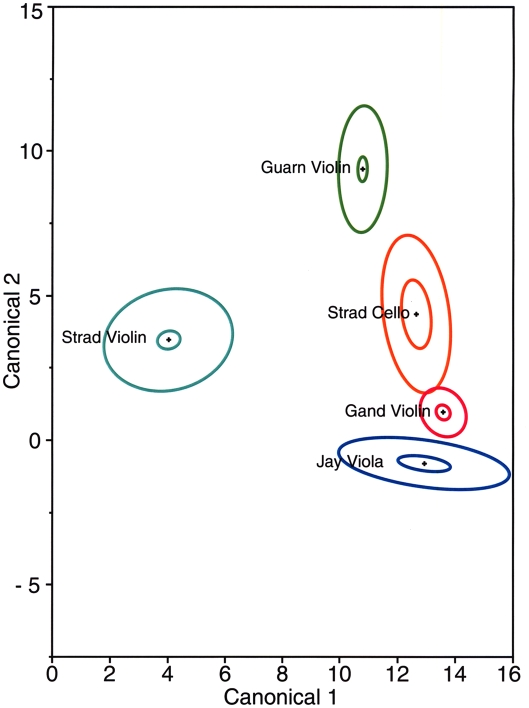
Canonical plot of musical instruments. The results of discriminant analysis are shown for the violin and cello of Stradivari, the early period Guarneri violin, the violin of Gand-Bernardel and the viola of Jay. The small ellipses represent the area of 95% confidence; the large ellipses contain 50% of the measurements.

The complete set of elemental analysis data of all musical instruments and commercial wood samples that were subjected to statistical analysis can be found in the Supplemental Information (Table S1). The results of the multivariate statistical analysis are shown in two figures which depict the clusters of data in elliptical forms, for each sample a small one and a large one. Figure 7 shows the separation of the mineral clusters from the woods taken from Stradivarius, Guarneri, Gand-Bernardel, and Jay instruments. In each instance, the small centroid ellipses represent 95%confidence for the mean chemical content, and the large ellipses contain about 50% of the measurements. The separation of the Stradivari violin and the Guarneri violin clusters is very obvious, and they are also well distanced from the Stradivarius cello, the Henry Jay viola and the Gand-Bernardel violin. Fig. 7 shows a small degree of overlap between the Stradivari cello and the Gand-Bernardel violin.

**Figure 8:**
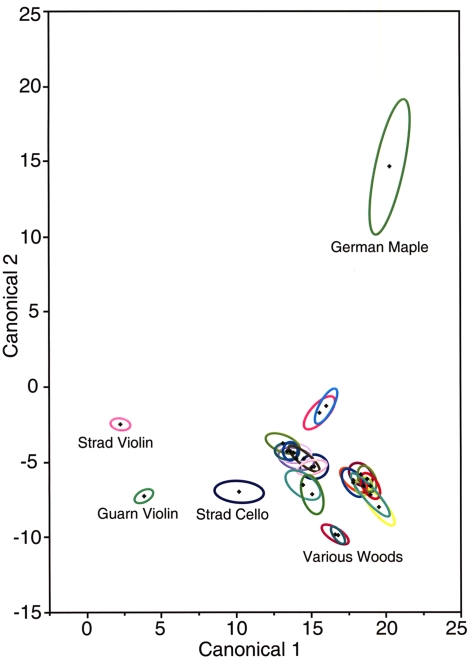
Canonical plot of Cremona instruments and commercial woods. The results of discriminant analysis are shown on the violin and cello of Stradivari, the early period Guarneri violin, and a variety of commercial woods. The small ellipses represent the area of 95%confidence.

Figure 8 shows 95% confidence ellipses for the mean chemical content for the Stradivarius and Guarneri instruments as well as for various commercial woods. Again, the plots used canonical axes which were designed to separate the instruments and woods as much as possible. These axes are different from those of Figure 7 as a separate optimization was done using the same method. The centroids for Stradivarius, Guarneri instruments are very different from the commercial woods with the exception of the Stradivarius cello.

## Discussion

The above results, however limited in the numbers of the precious specimens, offer the first real insight into the condition of the wood used by the two greatest violinmakers, Stradivari and Guarneri. It would be highly unlikely that out of the approx. 800 extant Stradivari and Guarneri instruments we would have come across the only four that possess unusual material compositions. All four samples from the instruments of these two masters have provided evidence of chemical manipulations. This conclusion rests foremost on the presence of a few minerals which are not known to exist in detectable quantities in natural woods. Most significant is the finding of borate in the violin and the cello of Stradivari and also in the early period violin of Guarneri. The latter also contained a most unexpected material, crystals of ZrSiO_4_. The late period Guarneri did not contain borate, but it had a small amount of the mineral fluorite, CaF_2_. The uptake of salts and especially the insoluble particles, like BaSO_4_, SiO_2_, and CaF_2_, may vary slightly according to the distribution of the vessels and the curvature of the violin surface. Unfortunately, we did not have the luxury of getting samples from different parts of the same instrument.

The four Cremona maple samples also differ from the others with regards to the amounts and the ratios of the common minerals of the wood. This seems to be the case if we examine the Na, K, Ca and SO_3_ contents listed in Tables 1 and 2. When considering a large number of the minerals, it requires statistical methods to assign varying degrees of significance to the differences. The results of the statistical evaluation relying on the method of discriminant analysis, shown in Figures 7 and 8, provide a graphic representation of the differences between the individual instruments on one hand and between the instruments and commercial woods, on the other hand. According to this analysis, there is no commercial wood that comes even remotely close to the violins of Stradivari and Guarneri, while the cello of Stradivari has some similarities to one of the commercial woods, the Slovenian wood. The commercial woods themselves reveal a considerable degree of variation which may be due to differences in the soil minerals and surface contaminations. It should be noted that the suppliers could not rule out the possibility that the logs could have been sprayed with fungicides.

An important insight one can gain from the above body of evidence is the apparent absence of a specific formula that would have been commonly used in Cremona. The chemicals found in the violin and the cello of Stradivari have quite different proportions. Perhaps this could be explained by the size difference of these instruments. The large plank of wood for the cello would have been hard to boil in a large vat, and its penetration by chemicals would have been limited. There are several differences between the two Guarneri violins, one of which relates to the borate and fluorite content. If we consider the primitive state of chemistry in the early 18^th^ century, it would not be surprising that these two minerals could have been misidentified. Under those circumstances, it could have been almost impossible to reproduce the intended chemical mixture over the years.

If we scrutinize the extraneous chemicals found in the Cremonese woods, there seems to be a probable intent in their being compounded in this particular fashion. They might have been intended for the purpose of wood preservation. The practice of employing borax as an insecticide and fungicide is so ancient that one cannot assign credit for its discovery [24]. It is interesting that similar uses of soluble fluorides and even their combination with borax can be found in current literature [25]. Other salts commonly sprayed on trees, like those of Zn, Cr, Cu and Fe, could have been included by design or as contaminants.

Apart from the above conclusions, one can make only educated guesses about the actual method of wood treatment in Cremona that harkens back to the alchemist practices of Paracelsus [26], who used an undefined mix of “salt of gems” for hardening of the wood. We can get now a glimpse into the possible composition of such mixture when inspecting the data of Table 1. The Cremona “salt of gems” could have contained crushed crystals of calcite, gypsum, barite, fluorite, and quartz, in addition to some water-soluble salts like borax and the sulfates of Zn, Cu, Cr and Fe. Possibly, the plank of wood could have been immersed in an aqueous slurry of the above materials and most likely boiled—a guaranteed way to kill the woodworm. It is not possible to ascertain if there had been only one such treatment or more, and whether the excessive soluble salt was extracted with ordinary water. One can assume that a thick slurry of minerals could have been also applied directly to the surfaces of the carved instruments as fillers. The fine particles could have penetrated into the interior of the maple mainly via the vessels [27] and a limited number of extracellular openings, and hence they could be found only sporadically in our ash samples. One cannot rule out the possibility that an alcoholic solution of chimney soot was also applied to the wood, this having been the practice of the last great Cremona trained violin-maker, G.B. Guadagnini [23].

Regrettably, our work had to be restricted to maple samples from the backs of instruments. There is much less spruce in the violin than maple, and we were unable to acquire samples in sufficient amounts for this set of analyses. The preliminary results on the few spruce samples we examined [11] by neutron-activation analysis suggested a higher concentration of minerals than in natural spruce. There would be no reason to assume that the spruce for musical instruments was not preserved the same way as the maple.

The question whether the minerals found in the four Cremona instruments were responsible for the previously reported degradation of the organic matrix of the wood [13] cannot be answered with sufficient confidence, but they could be an important factor. The Stradivarius cello exhibits the least deviation from the natural mineral composition, and it also shows the least damage to its hemicellulose and lignin components. Similarly, the samples from the instruments of Gand-Bernardel and Jay did not deviate much from natural wood in either their organic components or their mineral content. Since the violins of Stradivari and Guarneri revealed the greatest damage to their wood while their mineral composition differed substantially both from commercial wood and from each other, it would be hard to assert which of the chemicals were responsible for the changes. Sea salt by itself was shown to cause degradation when sprayed on live trees; salts of Fe, Cu, Cr, Sn, and Zn were also known to cause degradation [28]. An assortment of soluble salts, some of them being also oxidizing agents, were most likely involved in the degradation of the Cremona maples.

Our findings may have far-reaching implications for the direction of violin research since they seem to validate the assumptions of the minority view of what is essential for the production of fine violins. Much of the work during the last two centuries was focused on the geometry and the acoustical adjustment of the various elements of the violin. The selection of wood with the highest possible stiffness was considered essential, but the idea of using anything but unadulterated, natural wood has been an anathema to most violin-makers and researchers. Soaking of the wood and the addition of mineral preservatives appeared counter-intuitive since they had a negative effect on the elasticity modulus [2], [29]. However, one can also register a number of beneficial physical changes that would accompany the hypothetical Cremonese treatment of the wood based on research from several laboratories.

Breaking with the conventional practices, during the years since 1975, Nagyvary and his associates have built approximately 200 violins from wood that was treated by a variety of chemicals, including borax, microorganisms and hemicellulase enzymes [30]. Undoubtedly, other violinmakers have followed suit. These treatments were designed to lower the hemicellulose content, and therefore, also the moisture content and the density [30]–[32]. The increase of porosity and permeability of chemically and microbially treated wood was noted by several investigators [33], [11]. It was also reported by Bucur [34] that boiling of the wood led to a decrease of its density which is beneficial for the radiation of the sound. According to Nagyvary [11], the full benefit of the aqueous wood treatment includes an increase of porosity and permeability, which is apparent on staining of the wood and, especially, the early wood.

Two recent reports published this year (2008) seem to have a strong bearing on our conclusions concerning the Cremonese wood treatment. Schwarze *et al.* [35] employed specific fungi for the controlled degradation of Norway spruce and sycamore and achieved a substantial reduction of density and an increase of permeability, especially in the early wood. While this treatment reduced the elasticity modulus of the wood, nevertheless it improved its acoustical properties with respect to the radiation ratio. Even more recently, Stoel and Borman [36] applied computed tomography to study the densities of early wood and late wood in five Cremona violins and eight violins made by distinguished contemporary makers. With one exception beyond the maker's control, the modern violins were made of natural wood. The density differential between the early wood and the late wood was found significantly less in the Cremona violins than in the modern violins. The authors did not offer a convincing interpretation of their finding, but they assumed it could have been an outcome of an aqueous treatment of the wood.

The limitations of our study are obvious: our measurements and statistics encompassed only materials from a square inch of surface from a few musical instruments. In an ideal world of scientist-collector cooperation, one would like to have several areas of the same instrument sampled, and one would like to have several samples from all creative periods of Stradivari and Guarneri. It is our hope that others would now be attracted to this promising field of research and would be more successful than we were in acquiring precious samples.

In summary, several lines of evidence suggest that the wood of the great Italian masters of Cremona was chemically and physically different from those of the later makers. These new insights strengthen the case for the postulated chemical/material paradigm of the Cremona violin [37], [38], which posits chemical manipulations as a *sine-qua-non* towards the successful reproduction of the old standards. It follows then that natural wood may not be a suitable material if the goal is to make violins with the old Cremona-type tone. This recognition may instigate a major change in how the commercial wood should be processed and also in the state of the art of violin-making.

## Supporting Information

Table S1 Data for multivariate discriminant analysis. The full set of data included 95×12 values for the Stradivarius violin, 75×12 for the early Guarneri and 30×12 for the rest, with the exception of the German maple which had only 15×12 data points. The data set from the pellets from each musical instrument was analyzed in its entirety as one group, while the commercial woods were analyzed as sets of 15. Abbreviations: Guarn: the early Guarneri violin; Strad: Stradivari; StrCello: Stradivari cello; Gand: Gand-Bernardel violin. The following commercial wood ashes were analyzed: Bosn2M1 and Bosn2M2 are 2 groups of 15 sites each from the Bosnian tree no. 2; similarly, Bosn3M1, Bosn3M2 are 2 groups of 15 from the Bosnian tree no. 3 tree and so on up to Bosn6M1 and Bosn6M2; ChinaM1 and ChinaM2 are 2 groups of 15 sites from the ash pellet of one Chinese maple; GerMaple has only15 sites from the pellet of the German maple; slven1-ht and slven1-sp are groups of 15 sites from the heartwood and sapwood of a Slovenian maple, and slven2-ht and slven2-sp are 2 groups of 15 from the same board but a deeper layer of wood; slvk1-ht and slvk1-sp are 15 sites each from the heartwood and sapwood of a Slovakian maple, and slvk2-ht and slvk2-sp represent sites from the same board but a deeper layer; slvk1995-1 and slvk1995-2 designate 2 groups of 15 sites from a different Slovakian maple board obtained in 1995.
(1.90 MB DOC)
