# Supplementary material for: Mineral Preservatives in the Wood of Stradivari and Guarneri
Source: PLoS One. 2009 Jan 22;4(1):e4245. doi: 10.1371/journal.pone.0004245 (PMC2621340; doi:10.1371/journal.pone.0004245)
Supplement: Table S1 — Data for multivariate discriminant analysis. The full set of data included 95×12 values for the Stradivarius violin, 75×12 for the early Guarneri and 30×12 for the rest, with the exception of the German maple which had only 15×12 data points. The data set from the pellets from each musical instrument was analyzed in its entirety as one group, while the commercial woods were analyzed as sets of 15. Abbreviations: Guarn: the early Guarneri violin; Strad: Stradivari; StrCello: Stradivari cello; Gand: Gand-Bernardel violin. The following commercial wood ashes were analyzed: Bosn2M1 and Bosn2M2 are 2 groups of 15 sites each from the Bosnian tree no. 2; similarly, Bosn3M1, Bosn3M2 are 2 groups of 15 from the Bosnian tree no. 3 tree and so on up to Bosn6M1 and Bosn6M2; ChinaM1 and ChinaM2 are 2 groups of 15 sites from the ash pellet of one Chinese maple; GerMaple has only15 sites from the pellet of the German maple; slven1-ht and slven1-sp are groups of 15 sites from the heartwood and sapwood of a Slovenian maple, and slven2-ht and slven2-sp are 2 groups of 15 from the same board but a deeper layer of wood; slvk1-ht and slvk1-sp are 15 sites each from the heartwood and sapwood of a Slovakian maple, and slvk2-ht and slvk2-sp represent sites from the same board but a deeper layer; slvk1995-1 and slvk1995-2 designate 2 groups of 15 sites from a different Slovakian maple board obtained in 1995. (1.90 MB DOC) [file pone.0004245.s001.doc]

**Table S1. Data for multivariate discriminant analysis.**

| Instrument | Cl | Na2O | K2O | CaO | MgO | SiO2 | Al2O3 | P2O5 | SO3 | FeO | MnO | TiO2 |
| --- | --- | --- | --- | --- | --- | --- | --- | --- | --- | --- | --- | --- |
| Guarn Violin | 0.17 | 5.66 | 14.28 | 23.64 | 3.09 | 3.81 | 2.45 | 1.4 | 20.98 | 3.36 | 0.17 | 0.34 |
| Guarn Violin | 0.12 | 6.87 | 13.99 | 20.94 | 2.62 | 4.82 | 4.03 | 1.55 | 22.28 | 7.1 | 0.12 | 0.39 |
| Guarn Violin | 0.12 | 7.55 | 15.52 | 19.4 | 2.21 | 6.04 | 4.64 | 1.04 | 20.73 | 6.47 | 0.14 | 0.37 |
| Guarn Violin | 0.17 | 5.8 | 12.56 | 20.94 | 2.43 | 5.86 | 4.31 | 1.48 | 22.83 | 8.44 | 0.14 | 0.48 |
| Guarn Violin | 0.12 | 11.51 | 11.73 | 23.88 | 3.57 | 2.39 | 2.1 | 1.39 | 11.99 | 2.16 | 0.22 | 0.25 |
| Guarn Violin | 0.15 | 6.78 | 12.91 | 24.86 | 2.94 | 3.54 | 2.04 | 1.63 | 20.92 | 3.26 | 0.11 | 0.29 |
| Guarn Violin | 0.24 | 5.78 | 12.44 | 25.13 | 3.84 | 2.95 | 2.7 | 2.03 | 23.77 | 3.92 | 0.15 | 0.36 |
| Guarn Violin | 0.19 | 7.63 | 12.89 | 8.58 | 1.66 | 16.68 | 12.55 | 0.86 | 10.01 | 15.45 | 0.82 | 1.29 |
| Guarn Violin | 0.16 | 5.45 | 14.66 | 22.73 | 3.07 | 3.72 | 2.8 | 2.21 | 23.59 | 4.36 | 0.23 | 0.13 |
| Guarn Violin | 0.16 | 5.47 | 12.07 | 23.06 | 2.38 | 5.77 | 4.34 | 2.19 | 20.95 | 7.52 | 0.16 | 0.43 |
| Guarn Violin | 0.13 | 5.13 | 13.67 | 22.22 | 2.15 | 4.22 | 3.29 | 1.37 | 27.47 | 4.84 | 0.1 | 0.36 |
| Guarn Violin | 0.13 | 6.2 | 15.56 | 24.89 | 2.83 | 1.99 | 1.64 | 0.89 | 22.97 | 2.2 | 0.14 | 0.1 |
| Guarn Violin | 0.23 | 5.64 | 13.38 | 14.73 | 2.55 | 10.03 | 8.9 | 1.78 | 13.1 | 14.68 | 0.21 | 0.67 |
| Guarn Violin | 0.11 | 9.83 | 10.87 | 24.08 | 4.89 | 3.93 | 2.43 | 2.68 | 14.63 | 3.59 | 0 | 0.16 |
| Guarn Violin | 0.16 | 5.14 | 9.63 | 16.68 | 1.71 | 14.01 | 8.17 | 1.42 | 17.34 | 12.59 | 0.14 | 1.49 |
| Guarn Violin | 0.19 | 7.23 | 12.77 | 22.11 | 3.03 | 5.18 | 4.45 | 1.59 | 19.11 | 6.59 | 0.12 | 0.26 |
| Guarn Violin | 0.27 | 4.67 | 11.01 | 29.78 | 3.53 | 1.88 | 1.91 | 2.09 | 20.87 | 2.2 | 0.14 | 0.15 |
| Guarn Violin | 0.13 | 10.08 | 15.34 | 21.83 | 2.2 | 1.66 | 1.22 | 1.73 | 24.18 | 1.29 | 0 | 0.11 |
| Guarn Violin | 0.13 | 4.54 | 11.66 | 15.59 | 1.2 | 10.26 | 8.26 | 0.93 | 21.93 | 13.34 | 0.2 | 1.06 |
| Guarn Violin | 0.21 | 6.56 | 15.29 | 19.89 | 3.45 | 4.8 | 3.95 | 1.27 | 19.42 | 5.39 | 0 | 0.59 |
| Guarn Violin | 0.13 | 6.82 | 15.6 | 23.02 | 3.58 | 2.91 | 2.02 | 1.83 | 19.34 | 3.84 | 0.11 | 0.14 |
| Guarn Violin | 0.15 | 7.58 | 13.46 | 17.91 | 2.6 | 8.03 | 6.72 | 1.53 | 16.13 | 9 | 0.09 | 0.35 |
| Guarn Violin | 0.17 | 5.13 | 12.25 | 11.9 | 0.84 | 12.36 | 10.65 | 0.99 | 14.83 | 17.7 | 0.13 | 0.96 |
| Guarn Violin | 0.13 | 10.56 | 15.64 | 22.4 | 3.57 | 1.46 | 1.18 | 2.74 | 19.46 | 1.63 | 0.01 | 0.01 |
| Guarn Violin | 0.12 | 6.24 | 14.51 | 21 | 2.41 | 5.38 | 4.63 | 1.32 | 21.7 | 5.98 | 0.17 | 0.14 |
| Guarn Violin | 0.17 | 5.51 | 13.84 | 21.24 | 3.26 | 5.89 | 3.84 | 1.58 | 22.51 | 6.33 | 0.1 | 0.42 |
| Guarn Violin | 0.14 | 7.05 | 12.48 | 20.77 | 3.21 | 4.67 | 3.9 | 1.8 | 17.7 | 5.95 | 0.14 | 2.76 |
| Guarn Violin | 0.2 | 5.93 | 12.55 | 19.59 | 2.32 | 8.49 | 5.7 | 1.64 | 18.79 | 8.37 | 0.15 | 0.45 |
| Guarn Violin | 0.15 | 6.54 | 13.25 | 19.63 | 2.15 | 6.89 | 5.54 | 1.38 | 17.94 | 10.7 | 0.12 | 0.74 |
| Guarn Violin | 0.16 | 6.05 | 18.24 | 22.59 | 1.56 | 1.11 | 1.16 | 1.03 | 21.31 | 4 | 0.04 | 0.01 |
| Guarn Violin | 0.14 | 6.53 | 13.43 | 22.26 | 2.66 | 8.35 | 8 | 2.53 | 13.66 | 1.15 | 0.11 | 0.34 |
| Guarn Violin | 0.17 | 5.15 | 12.55 | 27.22 | 5.55 | 2.08 | 1.66 | 1.68 | 19.84 | 2.88 | 0.11 | 0.2 |
| Guarn Violin | 0.18 | 5.48 | 13.45 | 24.44 | 3.54 | 2.78 | 2.12 | 1.89 | 19.56 | 3.01 | 0.13 | 0.18 |
| Guarn Violin | 0.15 | 7.3 | 11.03 | 25.1 | 5.7 | 3.33 | 2.65 | 2.13 | 17.62 | 3.35 | 0.08 | 0.19 |
| Guarn Violin | 0.1 | 7.88 | 12.71 | 20.1 | 1.89 | 5.08 | 4.16 | 1.63 | 21.6 | 6.28 | 0.13 | 0.29 |
| Guarn Violin | 0.11 | 3.89 | 9.42 | 19.17 | 1.02 | 9.48 | 7.78 | 1.01 | 24.29 | 13.12 | 0.19 | 0.67 |
| Guarn Violin | 0.14 | 6.99 | 12.83 | 18.16 | 2.68 | 7.37 | 5.7 | 1.58 | 18.39 | 10.19 | 0.2 | 0.41 |
| Guarn Violin | 0.17 | 6.19 | 17.08 | 23.53 | 2.5 | 1.64 | 1.29 | 1.11 | 22.95 | 2.35 | 0.07 | 0.2 |
| Guarn Violin | 0.18 | 3.68 | 7.59 | 10.64 | 1.17 | 15.9 | 12.57 | 0.85 | 14.44 | 22.3 | 0.31 | 0.81 |
| Guarn Violin | 0.17 | 8.4 | 15.57 | 21.54 | 4.1 | 2.6 | 2.38 | 2.56 | 16.77 | 4.4 | 0.09 | 0.71 |
| Guarn Violin | 0.11 | 8.24 | 14.12 | 22.02 | 3.82 | 3.28 | 2.61 | 1.49 | 21.18 | 3.33 | 0.02 | 0.21 |
| Guarn Violin | 0.15 | 7.79 | 15.43 | 16.61 | 2.22 | 7.01 | 5.94 | 1.13 | 19.76 | 8.26 | 0.08 | 0.7 |
| Guarn Violin | 0.21 | 5.2 | 11.82 | 28.04 | 4.23 | 2.06 | 2.16 | 2.43 | 23.09 | 2.48 | 0.18 | 0.22 |
| Guarn Violin | 0.19 | 6.2 | 16.01 | 20.42 | 4.56 | 5.16 | 5.12 | 4.04 | 22.93 | 3.58 | 0.3 | 0.39 |
| Guarn Violin | 0.16 | 8.03 | 14.54 | 18.37 | 2.66 | 6.49 | 3.74 | 1.43 | 21.77 | 6.13 | 0.12 | 0.29 |
| Guarn Violin | 0.15 | 5.87 | 11.58 | 16.49 | 1.99 | 8.27 | 6.74 | 1.24 | 16.96 | 14.76 | 0.21 | 0.39 |
| Guarn Violin | 0.17 | 5.62 | 12.41 | 24.26 | 2.72 | 4.08 | 3.15 | 1.47 | 22.96 | 4.95 | 0.17 | 0.61 |
| Guarn Violin | 0.16 | 7.72 | 18.54 | 21.14 | 2.54 | 2.55 | 1.24 | 1.27 | 20.12 | 1.85 | 0.1 | 0.26 |
| Guarn Violin | 0.1 | 5.97 | 12.33 | 19.35 | 1.94 | 7.82 | 5.5 | 1.28 | 21.9 | 8.82 | 0.14 | 1.26 |
| Guarn Violin | 0.12 | 7.39 | 14.48 | 24.31 | 3.58 | 1.56 | 1.26 | 3.18 | 23.85 | 1.85 | 0.14 | 0.15 |
| Guarn Violin | 0.22 | 4.32 | 11.42 | 25.27 | 3.2 | 5.39 | 4.69 | 1.97 | 22.66 | 5.01 | 0.4 | 0.27 |
| Guarn Violin | 0.42 | 6.78 | 19.3 | 18.75 | 2.07 | 1 | 1.06 | 1.04 | 18.92 | 2.38 | 0 | 0.05 |
| Guarn Violin | 0.21 | 5.51 | 13.07 | 21.67 | 3.99 | 5.36 | 4.53 | 1.11 | 19.06 | 7.94 | 0.1 | 0.67 |
| Guarn Violin | 0.15 | 4.83 | 9.79 | 18.25 | 1.59 | 12.11 | 7.79 | 1.02 | 20.51 | 14.74 | 0.31 | 0.89 |
| Guarn Violin | 0.33 | 7.23 | 15.65 | 18.13 | 2.51 | 5.34 | 4.3 | 1.6 | 18.75 | 6.61 | 0.16 | 0.41 |
| Guarn Violin | 0.2 | 4.28 | 11.47 | 22 | 2.92 | 7.62 | 4.83 | 1.27 | 22 | 8.19 | 0.14 | 0.49 |
| Guarn Violin | 0.2 | 6.32 | 11.85 | 24.78 | 3.52 | 3.92 | 3.09 | 2.33 | 19.75 | 4.19 | 0.09 | 0.43 |
| Guarn Violin | 0.12 | 5.6 | 12.91 | 18.08 | 1.94 | 7.95 | 5.95 | 1.1 | 19.97 | 9.06 | 0.08 | 0.67 |
| Guarn Violin | 0.16 | 5.35 | 10.94 | 21.88 | 2 | 7.36 | 6.09 | 1.52 | 19.85 | 9.54 | 0.1 | 0.61 |
| Guarn Violin | 0.17 | 5.62 | 13.44 | 23.05 | 2.83 | 5.3 | 3.87 | 1.31 | 24.05 | 3.58 | 0.05 | 0.47 |
| Guarn Violin | 0.14 | 5.07 | 12.53 | 19.86 | 1.63 | 8.29 | 5.49 | 1 | 22.33 | 8.81 | 0.07 | 0.68 |
| Guarn Violin | 0.21 | 6.55 | 12.79 | 29.42 | 3.69 | 0.62 | 0.43 | 2.91 | 19.89 | 0.94 | 0.05 | 0.09 |
| Guarn Violin | 0.12 | 6.12 | 10.26 | 12.38 | 1.36 | 14.04 | 10.88 | 1.02 | 12.68 | 18.82 | 0.08 | 1.3 |
| Guarn Violin | 0.15 | 8.35 | 15.71 | 18.51 | 2.39 | 4.74 | 3.99 | 1.33 | 20.33 | 7.29 | 0.1 | 0.39 |
| Guarn Violin | 0.13 | 6.03 | 15.06 | 18.32 | 1.72 | 6.7 | 5.69 | 1.38 | 20.73 | 9.29 | 0.16 | 0.29 |
| Guarn Violin | 0.2 | 6.47 | 11.29 | 15.92 | 1.87 | 9.04 | 8.08 | 1.33 | 17.3 | 14.47 | 0.16 | 1.55 |
| Guarn Violin | 0.18 | 4.71 | 9.65 | 13.61 | 1.71 | 12.52 | 10.07 | 1.05 | 11.8 | 19.15 | 0.36 | 0.68 |
| Guarn Violin | 0.14 | 7.56 | 11.74 | 15.6 | 1.97 | 10.48 | 8.42 | 1.46 | 14.97 | 11.8 | 0.19 | 0.35 |
| Guarn Violin | 0.11 | 7.24 | 14.29 | 24.01 | 3.59 | 2.1 | 1.48 | 1.63 | 21.85 | 3.62 | 0.07 | 0.12 |
| Guarn Violin | 0.11 | 8.56 | 12.39 | 24.89 | 3.82 | 2.69 | 2 | 2 | 17.42 | 2.96 | 0.1 | 0.33 |
| Guarn Violin | 0.13 | 6.4 | 12.7 | 15.85 | 2.27 | 10.51 | 7.57 | 1.73 | 15.49 | 11.97 | 0.19 | 0.71 |
| Guarn Violin | 0.1 | 6.51 | 12.6 | 19.24 | 2.5 | 7.86 | 6.38 | 1.64 | 15.95 | 9.62 | 0.19 | 0.5 |
| Guarn Violin | 0.11 | 6.12 | 14.17 | 29.33 | 3.69 | 0.82 | 0.57 | 2.17 | 17.33 | 0.69 | 0.09 | 0.11 |
| Guarn Violin | 0.1 | 4.18 | 12.31 | 23.66 | 1.47 | 5.87 | 4.51 | 1.12 | 25.54 | 6.68 | 0.14 | 0.34 |
| Guarn Violin | 0.16 | 6.74 | 15.48 | 16.84 | 2.25 | 7.53 | 6.23 | 0.99 | 20.15 | 9.15 | 0.18 | 0.76 |
| Strad Violin | 0.063 | 2.209 | 2.356 | 0.08 | 1.167 | 58.601 | 1.157 | 0.607 | 9.285 | 1.548 | 0.012 | 0 |
| Strad Violin | 3.014 | 5.334 | 8.477 | 4.46 | 2.28 | 17.289 | 0.36 | 0.747 | 14.671 | 0.405 | 0 | 0 |
| Strad Violin | 6.894 | 6.612 | 13.522 | 4.654 | 2.902 | 12.493 | 2.045 | 1.252 | 11.704 | 1.746 | 0.042 | 0 |
| Strad Violin | 2.333 | 9.017 | 13.866 | 7.089 | 3.403 | 13.645 | 2.251 | 1.36 | 15.254 | 1.3 | 0.052 | 0 |
| Strad Violin | 2.17 | 11.052 | 9.106 | 4.018 | 0.567 | 47.892 | 0.091 | 0.825 | 1.732 | 0.189 | 0 | 0.026 |
| Strad Violin | 12.921 | 18.319 | 11.903 | 9.793 | 8.57 | 2.417 | 0.546 | 1.344 | 7.018 | 0.765 | 0.008 | 0.026 |
| Strad Violin | 1.216 | 8.714 | 8.696 | 5.13 | 2.146 | 10.818 | 0.313 | 1.073 | 14.446 | 0.242 | 0.065 | 0 |
| Strad Violin | 19.92 | 12.755 | 31.862 | 4.48 | 1.096 | 0.028 | 0.066 | 0.375 | 5.831 | 0.162 | 0.056 | 0.034 |
| Strad Violin | 12.595 | 16.974 | 23.048 | 8.614 | 1.924 | 0.94 | 0.342 | 1.191 | 6.733 | 0.785 | 0.132 | 0.129 |
| Strad Violin | 15.26 | 17.665 | 23.948 | 7.246 | 2.103 | 0.525 | 0.369 | 1.105 | 4.215 | 0.214 | 0.032 | 0 |
| Strad Violin | 15.698 | 18.382 | 22.832 | 6.58 | 2.162 | 0.85 | 0.208 | 0.761 | 3.857 | 0.338 | 0.076 | 0 |
| Strad Violin | 17.555 | 17.681 | 25.773 | 4.671 | 1.662 | 0.331 | 0.155 | 0.728 | 3.633 | 0.263 | 0.036 | 0 |
| Strad Violin | 16.68 | 19.297 | 24.393 | 5.344 | 1.436 | 0.408 | 0.077 | 0.642 | 3.791 | 0.182 | 0.016 | 0.051 |
| Strad Violin | 16.765 | 16.851 | 25.866 | 6.223 | 1.449 | 0.254 | 0.066 | 1.048 | 3.461 | 0.105 | 0.004 | 0.043 |
| Strad Violin | 14.979 | 14.74 | 24.027 | 6.889 | 2.515 | 0.596 | 0.799 | 0.655 | 4.992 | 0.19 | 0.012 | 0.086 |
| Strad Violin | 11.17 | 21.218 | 17.146 | 8.662 | 4.964 | 0.768 | 0.424 | 0.96 | 4.599 | 0.402 | 0.08 | 0 |
| Strad Violin | 4.995 | 9.98 | 16.14 | 15.729 | 1.833 | 5.107 | 1.221 | 1.188 | 9.042 | 4.026 | 0.629 | 0.488 |
| Strad Violin | 8.249 | 14.367 | 19.947 | 11.722 | 2.552 | 1.714 | 0.79 | 1.766 | 8.638 | 0.806 | 0.044 | 0.104 |
| Strad Violin | 4.389 | 14.8 | 15.633 | 12.232 | 2.972 | 0 | 0.015 | 0.159 | 11.262 | 0.891 | 0.043 | 0.051 |
| Strad Violin | 12.071 | 18.829 | 19.66 | 8.137 | 2.764 | 0.808 | 0.495 | 0.818 | 5.372 | 0.297 | 0.044 | 0.043 |
| Strad Violin | 8.154 | 15.474 | 18.275 | 9.761 | 4.346 | 3.715 | 0.283 | 1.359 | 6.863 | 0.588 | 0.096 | 0.009 |
| Strad Violin | 9.866 | 19.204 | 15.733 | 9.011 | 6.19 | 1.405 | 0.317 | 1.373 | 4.017 | 0.598 | 0.06 | 0.043 |
| Strad Violin | 10.543 | 19.579 | 18.513 | 8.525 | 3.461 | 0.99 | 0.13 | 0.976 | 5.274 | 0.364 | 0 | 0 |
| Strad Violin | 5.831 | 12.608 | 16.357 | 9.913 | 4.037 | 6.779 | 1.119 | 1.759 | 7.094 | 0.694 | 0.048 | 0.146 |
| Strad Violin | 5.265 | 9.992 | 7.954 | 15.684 | 9.352 | 16.803 | 0.085 | 0.986 | 2.255 | 0.283 | 0.033 | 0.052 |
| Strad Violin | 4.389 | 13.232 | 14.876 | 12.528 | 4.273 | 0 | 0 | 0.088 | 11.912 | 0.405 | 0.103 | 0.077 |
| Strad Violin | 4.961 | 10.518 | 14.76 | 14.637 | 1.556 | 7.1 | 1.202 | 1.512 | 8.717 | 2.173 | 0.133 | 0.095 |
| Strad Violin | 9.854 | 21.398 | 17.18 | 8.406 | 2.747 | 0 | 0.006 | 0.011 | 6.651 | 0.121 | 0.076 | 0.009 |
| Strad Violin | 6.028 | 11.454 | 18.412 | 10.402 | 1.896 | 0 | 0.064 | 0.602 | 5.903 | 0.049 | 0.035 | 0 |
| Strad Violin | 12.714 | 19.97 | 18.978 | 7.454 | 2.086 | 0.281 | 0.015 | 0.611 | 4.25 | 0.097 | 0.004 | 0 |
| Strad Violin | 15.409 | 15.203 | 24.013 | 6.109 | 1.353 | 0.141 | 0.045 | 1.063 | 3.33 | 0.178 | 0.016 | 0 |
| Strad Violin | 12.623 | 11.906 | 23.208 | 7.211 | 2.76 | 0.079 | 0.061 | 0.469 | 7.425 | 0.467 | 0.1 | 0.026 |
| Strad Violin | 10.33 | 19.242 | 18.093 | 7.584 | 2.765 | 0.556 | 0.213 | 0.967 | 5.191 | 0.622 | 0.032 | 0.112 |
| Strad Violin | 10.274 | 18.983 | 17.775 | 8.044 | 2.826 | 0.481 | 0.12 | 0.951 | 5.19 | 0.844 | 0.048 | 0 |
| Strad Violin | 1.654 | 10.484 | 14.584 | 12.493 | 1.926 | 6.24 | 1.59 | 2.44 | 10.215 | 3.2 | 0.052 | 0.043 |
| Strad Violin | 7.289 | 16.876 | 15.957 | 11.053 | 3.339 | 1.641 | 0.395 | 1.594 | 5.914 | 0.876 | 0.084 | 0.069 |
| Strad Violin | 5.094 | 15.092 | 17.859 | 7.822 | 1.687 | 4.388 | 0.526 | 0.932 | 10.692 | 0.722 | 0 | 0.215 |
| Strad Violin | 9.797 | 18.048 | 17.06 | 8.765 | 2.015 | 2.375 | 0.125 | 0.704 | 4.967 | 0.141 | 0.056 | 0 |
| Strad Violin | 10.946 | 19.536 | 17.207 | 8.026 | 3.176 | 0.441 | 0.069 | 1.03 | 4.395 | 0.121 | 0.016 | 0.043 |
| Strad Violin | 8.832 | 19.993 | 15.559 | 7.863 | 4.189 | 0.723 | 0.398 | 1.075 | 4.71 | 0.509 | 0.028 | 0.009 |
| Strad Violin | 3.068 | 19.578 | 13.926 | 11.156 | 4.191 | 1.404 | 0.324 | 1.77 | 7.555 | 1.008 | 0.097 | 0.086 |
| Strad Violin | 9.621 | 19.29 | 15.772 | 8.966 | 3.7 | 0.484 | 0.083 | 1.258 | 4.753 | 0.184 | 0 | 0.06 |
| Strad Violin | 10.4 | 14.99 | 19.766 | 9.353 | 2.387 | 0.521 | 0.209 | 0.903 | 4.747 | 0.141 | 0.06 | 0 |
| Strad Violin | 10.528 | 15.976 | 19.207 | 8.737 | 2.298 | 0.529 | 0.167 | 0.82 | 4.608 | 0.223 | 0 | 0 |
| Strad Violin | 9.743 | 15.524 | 20.086 | 8.486 | 2.438 | 0 | 0 | 0.171 | 6.163 | 0.063 | 0.024 | 0 |
| Strad Violin | 10.479 | 10.354 | 21.592 | 6.335 | 3.617 | 1.68 | 0.337 | 0.866 | 4.579 | 1.135 | 0.02 | 0 |
| Strad Violin | 4.685 | 12.034 | 16.551 | 7.031 | 4.696 | 0.219 | 0.05 | 0.683 | 9.046 | 0.176 | 0.138 | 0.025 |
| Strad Violin | 7.937 | 18.371 | 15.564 | 8.825 | 3.507 | 0.851 | 0.354 | 1.135 | 4.7 | 0.471 | 0 | 0 |
| Strad Violin | 6.221 | 11.303 | 17.173 | 7.416 | 2.153 | 0.113 | 0.049 | 0.556 | 7.033 | 0.174 | 0.105 | 0 |
| Strad Violin | 4.12 | 14.178 | 15.291 | 11.771 | 3.123 | 2.219 | 0.807 | 1.905 | 6.492 | 0.587 | 0.077 | 0.086 |
| Strad Violin | 4.781 | 14.931 | 15.927 | 10.292 | 2.653 | 0.939 | 0.111 | 1.142 | 8.616 | 0.181 | 0 | 0.026 |
| Strad Violin | 9.547 | 11.98 | 17.261 | 9.996 | 3.29 | 1.531 | 0.677 | 1.011 | 4.578 | 0.429 | 0.092 | 0.456 |
| Strad Violin | 3.651 | 22.646 | 12.526 | 10.687 | 2.763 | 0.92 | 0.208 | 1.286 | 5.952 | 0.252 | 0.04 | 0.043 |
| Strad Violin | 2.272 | 21.855 | 12.049 | 9.987 | 3.716 | 0.756 | 0.096 | 1.443 | 8.148 | 0.261 | 0.069 | 0.026 |
| Strad Violin | 5.252 | 13.992 | 13.444 | 12.124 | 2.362 | 5.581 | 1.644 | 1.208 | 4.543 | 0.382 | 0.036 | 0.026 |
| Strad Violin | 11.428 | 7.113 | 22.069 | 8.928 | 1.439 | 1.679 | 0.36 | 1.077 | 5.889 | 0.888 | 0.14 | 0.026 |
| Strad Violin | 6.238 | 15.829 | 15.064 | 12.018 | 3.394 | 0.921 | 0.296 | 1.422 | 4.587 | 0.189 | 0.089 | 0.078 |
| Strad Violin | 3.936 | 10.875 | 14.776 | 13.275 | 3.846 | 1.154 | 0.686 | 1.897 | 8.594 | 0.466 | 0.101 | 0.156 |
| Strad Violin | 4.392 | 16.396 | 14.526 | 9.5 | 3.815 | 1.999 | 0.312 | 1.297 | 6.637 | 0.626 | 0.121 | 0.094 |
| Strad Violin | 6.164 | 18.728 | 14.211 | 8.991 | 2.499 | 0.594 | 0.11 | 1.137 | 6.648 | 0.126 | 0.097 | 0 |
| Strad Violin | 4.246 | 17.213 | 10.192 | 9.886 | 6.53 | 0.956 | 0.4 | 1.149 | 7.263 | 0.421 | 0.165 | 0 |
| Strad Violin | 3.763 | 20.601 | 10.908 | 12.469 | 5.373 | 0.417 | 0.032 | 1.481 | 3.831 | 0.036 | 0.02 | 0.017 |
| Strad Violin | 4.288 | 13.96 | 13.774 | 13.484 | 4.019 | 1.664 | 0.224 | 1.164 | 5.315 | 0.264 | 0.044 | 0 |
| Strad Violin | 5.903 | 18.672 | 14.736 | 8.874 | 2.204 | 0.713 | 0.207 | 0.88 | 6.913 | 0.3 | 0.036 | 0.06 |
| Strad Violin | 4.013 | 14.271 | 14.904 | 11.127 | 2.479 | 2.17 | 0.663 | 1.626 | 6.936 | 0.462 | 0.101 | 0.121 |
| Strad Violin | 2.957 | 26.159 | 9.212 | 10.343 | 3.06 | 0.403 | 0.068 | 1.747 | 4.985 | 0.131 | 0 | 0 |
| Strad Violin | 5.96 | 17.339 | 13.676 | 8.664 | 3.21 | 1.359 | 0.191 | 1.22 | 6.802 | 0.311 | 0.06 | 0.146 |
| Strad Violin | 5.434 | 18.652 | 12.53 | 11.556 | 3.443 | 0.835 | 0.251 | 1.296 | 3.919 | 0.146 | 0.048 | 0.043 |
| Strad Violin | 4.904 | 20.286 | 11.34 | 10.74 | 3.309 | 0.851 | 0.204 | 1.409 | 4.377 | 0.324 | 0.052 | 0 |
| Strad Violin | 2.535 | 20.199 | 9.521 | 10.18 | 4.33 | 1.295 | 0.456 | 1.135 | 5.711 | 1.337 | 0.101 | 0.009 |
| Strad Violin | 3.717 | 23.068 | 9.268 | 10.503 | 4.815 | 0.33 | 0.023 | 1.399 | 3.4 | 0.063 | 0.113 | 0 |
| Strad Violin | 2.708 | 14.759 | 14.288 | 8.052 | 3.413 | 0.913 | 0.211 | 1.471 | 10.265 | 0.316 | 0.089 | 0.043 |
| Strad Violin | 2.184 | 24.649 | 7.776 | 11.818 | 3.6 | 0.473 | 0.043 | 1.567 | 4.086 | 0.075 | 0.049 | 0 |
| Strad Violin | 2.935 | 20.359 | 10.856 | 10.884 | 3.078 | 0.921 | 0.128 | 1.234 | 5.586 | 0.138 | 0.057 | 0.06 |
| Strad Violin | 3.57 | 21.965 | 9.156 | 10.262 | 3.629 | 0.703 | 0.104 | 1.452 | 3.776 | 0.498 | 0.101 | 0.069 |
| Strad Violin | 3.771 | 25.774 | 10.141 | 8.084 | 1.602 | 0.314 | 0.03 | 1.202 | 3.269 | 0.039 | 0.069 | 0.086 |
| Strad Violin | 2.14 | 10.368 | 16.406 | 5.747 | 3.656 | 0.952 | 0.202 | 1.038 | 12.689 | 0.182 | 0.093 | 0 |
| Strad Violin | 2.981 | 13.521 | 11.228 | 12.38 | 3.479 | 1.56 | 0.513 | 1.715 | 5.358 | 0.569 | 0.089 | 0 |
| Strad Violin | 2.261 | 12.808 | 13.751 | 10.372 | 2.636 | 0.367 | 0.057 | 0.643 | 8.232 | 0.509 | 0.073 | 0.112 |
| Strad Violin | 5.257 | 10.932 | 13.602 | 13.025 | 2.392 | 1.117 | 0.149 | 1.432 | 3.717 | 0.373 | 0.077 | 0 |
| Strad Violin | 8.842 | 4.127 | 17.433 | 10.043 | 1.544 | 1.148 | 0.613 | 1.064 | 3.594 | 0.499 | 0.076 | 0 |
| Strad Violin | 5.007 | 21.51 | 9.271 | 5.472 | 2.027 | 0.609 | 0.372 | 0.5 | 2.536 | 0.083 | 0.04 | 0.017 |
| Strad Violin | 1.995 | 26.255 | 8.616 | 4.112 | 2.271 | 0.297 | 0.038 | 0.695 | 1.528 | 0.083 | 0.056 | 0.051 |
| Strad Violin | 5.387 | 0.001 | 13.393 | 11.378 | 0 | 0.026 | 0.002 | 0.015 | 9.467 | 1.131 | 0 | 0 |
| Strad Violin | 1.82 | 14.643 | 9.616 | 11.345 | 2.558 | 0 | 0.004 | 0.141 | 4.108 | 0.265 | 0.04 | 0.026 |
| Strad Violin | 5.18 | 0.027 | 16.218 | 12.209 | 0.288 | 0 | 0.002 | 0.845 | 5.532 | 0.141 | 0.024 | 0.018 |
| Strad Violin | 1.574 | 19.795 | 4.748 | 5.905 | 0.283 | 1.154 | 0.539 | 0.593 | 2.742 | 0.684 | 0.024 | 0.085 |
| Strad Violin | 8.079 | 0 | 13.791 | 8.811 | 0 | 0 | 0 | 0.019 | 4.431 | 0.264 | 0.064 | 0 |
| Strad Violin | 6.277 | 0.001 | 13.294 | 10.385 | 0 | 0 | 0.001 | 0 | 3.664 | 0.284 | 0.048 | 0 |
| Strad Violin | 0.603 | 15.682 | 2.621 | 1.842 | 7.095 | 0.979 | 0.273 | 1.228 | 0.087 | 0.491 | 0.093 | 0.067 |
| Strad Cello | 0.763 | 3.78 | 20.786 | 25.208 | 7.135 | 1.014 | 0.183 | 2.988 | 7.771 | 0.154 | 0.105 | 0.045 |
| Strad Cello | 0.535 | 4.194 | 20.17 | 24.959 | 8.65 | 1.508 | 0.505 | 4.845 | 11.173 | 0.664 | 0.163 | 0.055 |
| Strad Cello | 0.73 | 3.597 | 18.052 | 25.705 | 8.432 | 0.972 | 0.119 | 3.395 | 14.892 | 0.139 | 0.119 | 0.02 |
| Strad Cello | 1.116 | 6.887 | 10.236 | 29.196 | 11.622 | 1.428 | 0.35 | 4.66 | 10.786 | 0.2 | 0.155 | 0.025 |
| Strad Cello | 1.218 | 3.964 | 8.812 | 33.73 | 10.923 | 2.568 | 0.56 | 6.677 | 7.911 | 0.514 | 0.099 | 0.03 |
| Strad Cello | 0.612 | 3.388 | 12.325 | 24.994 | 9.012 | 6.832 | 3.878 | 4.747 | 11.548 | 2.302 | 0.159 | 0.56 |
| Strad Cello | 0.607 | 3.073 | 12.898 | 22.237 | 3.169 | 11.411 | 6.213 | 2.18 | 14.768 | 6.37 | 0.24 | 1.029 |
| Strad Cello | 1.104 | 3.686 | 10.273 | 31.526 | 6.336 | 4.967 | 1.977 | 3.066 | 12.558 | 1.61 | 0.219 | 0.297 |
| Strad Cello | 1.317 | 6.675 | 18.532 | 24.163 | 5.479 | 2.712 | 1.255 | 2.483 | 12.668 | 0.966 | 0.051 | 0.095 |
| Strad Cello | 1.549 | 5.786 | 11.526 | 28.172 | 9.593 | 4.936 | 2.018 | 4.893 | 8.391 | 0.727 | 0.103 | 0.106 |
| Strad Cello | 0.378 | 4.302 | 6.885 | 38.217 | 4.988 | 2.583 | 0.902 | 3.115 | 9.834 | 0.554 | 0.013 | 0 |
| Strad Cello | 0.512 | 3.523 | 10.347 | 34.35 | 9.33 | 2.644 | 0.658 | 4.077 | 8.494 | 0.318 | 0.123 | 0.116 |
| Strad Cello | 1.326 | 3.004 | 4.752 | 36.72 | 11.166 | 1.934 | 0.656 | 2.919 | 10.182 | 0.118 | 0.131 | 0 |
| Strad Cello | 1.231 | 6.181 | 3.967 | 43.518 | 0.961 | 5.585 | 2.837 | 0.855 | 4.967 | 0.545 | 0.038 | 0 |
| Strad Cello | 1.245 | 7.028 | 11.572 | 29.997 | 8.436 | 2.348 | 0.897 | 3.368 | 8.654 | 0.492 | 0.083 | 0.131 |
| Gand Violin | 0.212 | 5.505 | 9.516 | 26.488 | 5.054 | 1.299 | 0.712 | 2.595 | 3.608 | 16.423 | 0.165 | 0.05 |
| Gand Violin | 0.141 | 3.221 | 14.223 | 33.302 | 7.156 | 5.061 | 0 | 3.189 | 3.75 | 0.183 | 0.184 | 0 |
| Gand Violin | 0.146 | 2.724 | 13.609 | 34.294 | 7.167 | 0.953 | 0.288 | 4.024 | 5.145 | 0.318 | 0.204 | 0 |
| Gand Violin | 0.149 | 3.863 | 17.786 | 28.072 | 7.292 | 1.741 | 0.444 | 2.588 | 5.256 | 0.432 | 0.159 | 0.342 |
| Gand Violin | 0.16 | 4.776 | 16.573 | 28.544 | 6.821 | 2.73 | 0.413 | 3.305 | 3.939 | 0.297 | 0.175 | 0.053 |
| Gand Violin | 0.173 | 2.134 | 10.28 | 37.899 | 7.743 | 0.095 | 0.013 | 4.53 | 3.684 | 0.088 | 0.233 | 0.08 |
| Gand Violin | 0.168 | 2.13 | 9.488 | 39.887 | 6.153 | 0.114 | 0.017 | 4.071 | 3.974 | 0.088 | 0.143 | 0 |
| Gand Violin | 0.138 | 3.571 | 11.666 | 35.564 | 7.379 | 0.168 | 0.013 | 2.806 | 4.584 | 0.016 | 0.257 | 0.053 |
| Gand Violin | 0.142 | 3.816 | 16.651 | 30.428 | 6.475 | 0.627 | 0.163 | 3.356 | 3.676 | 0.341 | 0.159 | 0.009 |
| Gand Violin | 0.147 | 4.154 | 12.37 | 34.135 | 7.028 | 0.659 | 0.131 | 2.975 | 3.516 | 0.23 | 0.155 | 0 |
| Gand Violin | 0.158 | 4.876 | 15.061 | 31.605 | 5.66 | 0.191 | 0 | 2.841 | 5.19 | 0.02 | 0.167 | 0.044 |
| Gand Violin | 0.135 | 5.642 | 17.876 | 28.963 | 5.394 | 0.132 | 0.007 | 2.484 | 4.759 | 0.012 | 0.248 | 0 |
| Gand Violin | 0.188 | 4.251 | 13.455 | 33.786 | 6.912 | 0.135 | 0.056 | 3.075 | 3.481 | 0.028 | 0.22 | 0 |
| Gand Violin | 0.202 | 2.597 | 8.408 | 38.879 | 8.288 | 0.217 | 0.015 | 3.461 | 3.065 | 0.052 | 0.29 | 0.062 |
| Gand Violin | 0.09 | 1.634 | 7.494 | 39.145 | 9.362 | 0.065 | 0.009 | 4.152 | 2.98 | 0.052 | 0.208 | 0 |
| Gand Violin | 0.225 | 2.951 | 13.317 | 34.769 | 6.312 | 0.016 | 0.003 | 4.291 | 3.327 | 0.075 | 0.212 | 0.053 |
| Gand Violin | 0.152 | 4.383 | 9.115 | 35.965 | 8.474 | 0.057 | 0.015 | 3.816 | 2.813 | 0 | 0.249 | 0 |
| Gand Violin | 0.145 | 3.137 | 14.596 | 32.618 | 6.628 | 1.459 | 0.477 | 2.68 | 2.348 | 0.519 | 0.403 | 0.088 |
| Gand Violin | 0.114 | 3.167 | 8.846 | 36.854 | 9.143 | 0.034 | 0.022 | 3.987 | 2.693 | 0.032 | 0.147 | 0.018 |
| Gand Violin | 0.159 | 3.343 | 14.164 | 33.346 | 7.471 | 0 | 0.002 | 3.658 | 2.181 | 0.115 | 0.175 | 0 |
| Gand Violin | 0.158 | 2.74 | 15.184 | 33.015 | 6.564 | 0.244 | 0.037 | 2.662 | 3.836 | 0.099 | 0.151 | 0.035 |
| Gand Violin | 0.159 | 2.789 | 13.388 | 33.96 | 7.218 | 0 | 0.013 | 3.938 | 2.568 | 0.04 | 0.126 | 0.009 |
| Gand Violin | 0.104 | 3.165 | 15.636 | 32.309 | 6.462 | 0.079 | 0.006 | 3.24 | 3.135 | 0.059 | 0.183 | 0.018 |
| Gand Violin | 0.151 | 3.49 | 15.688 | 32.322 | 7.172 | 0.073 | 0.025 | 3.004 | 2.071 | 0.119 | 0.24 | 0.044 |
| Gand Violin | 0.153 | 4.055 | 16.033 | 30.936 | 6.099 | 0.183 | 0.033 | 3.088 | 3.442 | 0.151 | 0.155 | 0.088 |
| Gand Violin | 0.174 | 3.664 | 12.972 | 33.456 | 6.729 | 0.108 | 0.019 | 3.395 | 3.024 | 0.381 | 0.138 | 0.079 |
| Gand Violin | 0.132 | 2.771 | 12.003 | 35.049 | 7.424 | 0.019 | 0 | 3.695 | 2.331 | 0.071 | 0.269 | 0.018 |
| Gand Violin | 0.171 | 3.171 | 12.079 | 34.87 | 7.601 | 0.027 | 0.013 | 2.582 | 2.526 | 0.583 | 0.252 | 0 |
| Gand Violin | 0.167 | 3.36 | 15.776 | 31.833 | 6.539 | 0.127 | 0.018 | 2.96 | 2.247 | 0 | 0.159 | 0 |
| Gand Violin | 0.156 | 1.978 | 12.242 | 36.184 | 7.592 | 0.136 | 0.002 | 2.728 | 1.647 | 0 | 0.224 | 0 |
| Jay Viola | 0.216 | 4.135 | 18.918 | 27.293 | 5.27 | 5.322 | 1.012 | 5.607 | 3.658 | 0.611 | 0.228 | 0.044 |
| Jay Viola | 0.206 | 1.684 | 21.341 | 28.632 | 5.994 | 0.5 | 0.051 | 7.764 | 4.756 | 0.055 | 0.199 | 0 |
| Jay Viola | 0.348 | 5.108 | 23 | 20.078 | 4.321 | 7.427 | 2.582 | 3.477 | 2.449 | 0.97 | 0.105 | 0.235 |
| Jay Viola | 0.303 | 3.665 | 22.455 | 22.054 | 4 | 5.959 | 2.266 | 4.451 | 3.701 | 0.776 | 0.203 | 0.14 |
| Jay Viola | 0.254 | 3.786 | 21.113 | 27.908 | 6.432 | 1.027 | 0.334 | 6.464 | 2.272 | 0.091 | 0.187 | 0.018 |
| Jay Viola | 0.139 | 2.809 | 25.701 | 24.308 | 4.86 | 0.378 | 2.925 | 4.323 | 2.926 | 0.091 | 0.113 | 0 |
| Jay Viola | 0.254 | 2.66 | 24.503 | 26.94 | 6.078 | 0.777 | 0.022 | 4.105 | 2.637 | 0.122 | 0.154 | 0 |
| Jay Viola | 0.235 | 3.132 | 22.202 | 26.472 | 5.914 | 1.264 | 0.265 | 5.438 | 2.627 | 0.162 | 0.138 | 0 |
| Jay Viola | 0.274 | 2.931 | 25.646 | 25.589 | 5.971 | 0.241 | 0.028 | 3.893 | 2.699 | 0.043 | 0.162 | 0.07 |
| Jay Viola | 0.101 | 2.312 | 19.853 | 27.007 | 6.419 | 0.849 | 0.02 | 6.689 | 3.254 | 0.071 | 0.146 | 0 |
| Jay Viola | 0.328 | 4.2 | 22.075 | 25.229 | 5.249 | 1.589 | 0.368 | 5.055 | 2.586 | 0.194 | 0.191 | 0.061 |
| Jay Viola | 0.246 | 1.978 | 25.607 | 24.715 | 5.301 | 1.203 | 0.399 | 3.92 | 3.142 | 0.553 | 0.158 | 0.114 |
| Jay Viola | 0.156 | 3.783 | 15.158 | 29.098 | 6.488 | 0.938 | 0.03 | 7.704 | 3.453 | 0.131 | 0.106 | 0 |
| Jay Viola | 0.239 | 3.248 | 25.925 | 24.373 | 5.638 | 0.315 | 0.019 | 4.456 | 2.641 | 0.051 | 0.118 | 0 |
| Jay Viola | 0.33 | 2.929 | 23.043 | 25.041 | 5.796 | 1.374 | 0.327 | 4.704 | 2.897 | 0.166 | 0.235 | 0.07 |
| Jay Viola | 0.262 | 2.906 | 22.063 | 26.352 | 6.186 | 0.525 | 0.095 | 5.442 | 2.842 | 0.036 | 0.142 | 0.018 |
| Jay Viola | 0.208 | 1.716 | 23.794 | 26.418 | 6.14 | 0.228 | 0.03 | 4.447 | 3.548 | 0.075 | 0.101 | 0.053 |
| Jay Viola | 0.178 | 3.625 | 23.583 | 27.242 | 5.139 | 0.441 | 0.022 | 3.765 | 2.331 | 0.051 | 0.142 | 0 |
| Jay Viola | 0.229 | 4.601 | 22.843 | 23.618 | 5.322 | 1.335 | 0.347 | 4.427 | 3.088 | 0.217 | 0.183 | 0 |
| Jay Viola | 0.173 | 2.571 | 25.149 | 24.432 | 5.885 | 0.594 | 0.005 | 3.843 | 2.795 | 0.095 | 0.154 | 0 |
| Jay Viola | 0.229 | 1.519 | 24.804 | 26.057 | 5.538 | 0.21 | 0.014 | 4.767 | 2.316 | 0 | 0.207 | 0.044 |
| Jay Viola | 0.196 | 1.001 | 22.073 | 26.659 | 4.973 | 1.708 | 0.073 | 3.545 | 3.376 | 0.099 | 0.166 | 0.053 |
| Jay Viola | 0.2 | 0.778 | 23.082 | 28.06 | 4.961 | 0.469 | 0.024 | 3.518 | 2.493 | 0.11 | 0.13 | 0 |
| Jay Viola | 0.334 | 4.817 | 23.425 | 20.197 | 6.037 | 0.783 | 0.129 | 3.949 | 2.505 | 0.15 | 0.17 | 0.018 |
| Jay Viola | 0.304 | 5.848 | 23.814 | 20.718 | 3.933 | 0.501 | 0.114 | 3.351 | 3.212 | 0.087 | 0.085 | 0 |
| Jay Viola | 0.212 | 0.858 | 21.571 | 26.13 | 5.746 | 0.248 | 0.009 | 2.889 | 1.856 | 0 | 0.134 | 0 |
| Jay Viola | 0.22 | 4.684 | 20.106 | 21.264 | 4.141 | 1.837 | 0.669 | 2.943 | 2.769 | 0.209 | 0.114 | 0.053 |
| Jay Viola | 0.2 | 0.82 | 22.998 | 25.372 | 4.368 | 0.277 | 0 | 2.637 | 1.946 | 0.039 | 0.17 | 0.018 |
| Jay Viola | 0.218 | 4.939 | 21.695 | 19.081 | 4.361 | 2.095 | 0.275 | 2.939 | 1.707 | 0.103 | 0.194 | 0.044 |
| Jay Viola | 0.258 | 2.775 | 10.306 | 10.129 | 2.288 | 2.671 | 0.762 | 1.751 | 1.057 | 0.186 | 0.077 | 0.061 |
| Bosn2M1 | 0.07 | 0.83 | 19.26 | 22.92 | 21.03 | 0.04 | 0 | 2.54 | 1.9 | 0.09 | 0.25 | 0.04 |
| Bosn2M1 | 0.06 | 0.77 | 19.07 | 25.03 | 19.09 | 0.08 | 0.01 | 3.16 | 1.58 | 0.01 | 0.33 | 0 |
| Bosn2M1 | 0.08 | 0.65 | 15.36 | 26.64 | 20.23 | 0.21 | 0.01 | 3.62 | 1.73 | 0.01 | 0.42 | 0.04 |
| Bosn2M1 | 0.13 | 0.91 | 18.47 | 23.23 | 21.14 | 0.22 | 0 | 3.49 | 1.52 | 0.01 | 0.35 | 0.01 |
| Bosn2M1 | 0.11 | 0.89 | 17.15 | 25.98 | 20.66 | 0.14 | 0.02 | 2.63 | 1.77 | 0 | 0.4 | 0 |
| Bosn2M1 | 0.31 | 1.02 | 21.19 | 22.02 | 21.23 | 0.14 | 0.03 | 2.21 | 1.55 | 0 | 0.37 | 0 |
| Bosn2M1 | 0.24 | 0.92 | 20.25 | 19.26 | 22.43 | 0.1 | 0.02 | 2.54 | 1.07 | 0.01 | 0.25 | 0.01 |
| Bosn2M1 | 0.2 | 0.82 | 19.12 | 22.33 | 19.23 | 0.1 | 0 | 3.33 | 1.31 | 0.01 | 0.31 | 0 |
| Bosn2M1 | 0.04 | 0.84 | 18.79 | 22.82 | 19.61 | 0 | 0.01 | 2.7 | 1.06 | 0.04 | 0.37 | 0 |
| Bosn2M1 | 0.52 | 1.21 | 22.53 | 19.4 | 18.17 | 0 | 0 | 1.99 | 1.09 | 0.02 | 0.35 | 0 |
| Bosn2M1 | 0.13 | 0.75 | 18.86 | 23.49 | 18.11 | 0.11 | 0 | 2.27 | 1.36 | 0.03 | 0.37 | 0.04 |
| Bosn2M1 | 0.14 | 0.66 | 19.78 | 21 | 17.57 | 0.07 | 0 | 2.2 | 1.12 | 0.03 | 0.3 | 0 |
| Bosn2M1 | 0.09 | 0.7 | 17.25 | 22 | 20.14 | 0.13 | 0.03 | 2.72 | 1.46 | 0.08 | 0.29 | 0.04 |
| Bosn2M1 | 0.03 | 0.59 | 15.43 | 22.59 | 24.57 | 0.09 | 0.01 | 3.05 | 1.95 | 0 | 0.31 | 0.01 |
| Bosn2M1 | 0.04 | 0.68 | 16.87 | 23.39 | 20.06 | 0.32 | 0.01 | 3.35 | 1.44 | 0.06 | 0.44 | 0 |
| Bosn2M2 | 0.06 | 0.78 | 19.6 | 21.37 | 19.81 | 0.14 | 0 | 3.02 | 1.55 | 0 | 0.42 | 0 |
| Bosn2M2 | 0.08 | 0.59 | 15.97 | 23.67 | 23.23 | 0.04 | 0.02 | 3.47 | 1.89 | 0.06 | 0.45 | 0 |
| Bosn2M2 | 0.02 | 0.64 | 20.17 | 21.2 | 20.17 | 0.04 | 0 | 2.25 | 1.66 | 0.01 | 0.32 | 0.04 |
| Bosn2M2 | 0.04 | 0.68 | 20.09 | 21.34 | 19.42 | 0.1 | 0.05 | 2.01 | 1.56 | 0.13 | 0.32 | 0 |
| Bosn2M2 | 0.03 | 0.66 | 20.77 | 21.18 | 19.31 | 0 | 0.01 | 1.88 | 1.35 | 0.02 | 0.22 | 0.01 |
| Bosn2M2 | 0.05 | 0.44 | 12.29 | 26.79 | 21.59 | 0.12 | 0.01 | 4.5 | 1.49 | 0 | 0.39 | 0 |
| Bosn2M2 | 0.03 | 0.54 | 14.98 | 23.1 | 21.95 | 0.17 | 0.04 | 4.5 | 1.43 | 0.06 | 0.31 | 0 |
| Bosn2M2 | 0.11 | 0.55 | 15.35 | 24.23 | 20.59 | 0.32 | 0.01 | 3.58 | 1.27 | 0.02 | 0.32 | 0 |
| Bosn2M2 | 0.05 | 0.53 | 14.87 | 22.53 | 21.89 | 0.12 | 0.01 | 4.34 | 1.57 | 0.03 | 0.37 | 0 |
| Bosn2M2 | 0.09 | 0.78 | 20.34 | 19.9 | 20.24 | 0.01 | 0.33 | 2.3 | 1.31 | 0.02 | 0.23 | 0.04 |
| Bosn2M2 | 0.05 | 0.63 | 16.15 | 23.11 | 20.91 | 0.41 | 0 | 4.55 | 1.86 | 0.02 | 0.42 | 0 |
| Bosn2M2 | 0.13 | 0.71 | 18.29 | 20.45 | 19.3 | 0.07 | 0.01 | 2.46 | 1.13 | 0 | 0.29 | 0 |
| Bosn2M2 | 0.19 | 0.78 | 20.83 | 20.01 | 17.11 | 0.01 | 0 | 1.98 | 1.35 | 0.03 | 0.21 | 0.01 |
| Bosn2M2 | 0.24 | 0.71 | 18.63 | 21.64 | 18.87 | 0.06 | 0.02 | 2.91 | 0.94 | 0 | 0.23 | 0 |
| Bosn2M2 | 0.2 | 0.8 | 22.02 | 18.99 | 17.95 | 0.09 | 0 | 1.8 | 0.9 | 0.04 | 0.28 | 0.03 |
| Bosn3M1 | 0.12 | 0.71 | 11.72 | 30.18 | 18.17 | 0.19 | 0.05 | 2.95 | 1.81 | 0.07 | 0.42 | 0.01 |
| Bosn3M1 | 0.09 | 0.41 | 7.16 | 34.13 | 17.61 | 0.29 | 0.06 | 2.26 | 1.73 | 0.04 | 0.46 | 0.02 |
| Bosn3M1 | 0.12 | 0.62 | 9.54 | 31.01 | 18.86 | 0.56 | 0.08 | 3.88 | 1.74 | 0.08 | 0.4 | 0 |
| Bosn3M1 | 0.14 | 0.42 | 5.16 | 34.41 | 22 | 0.28 | 0 | 5.33 | 2.16 | 0 | 0.48 | 0 |
| Bosn3M1 | 0.13 | 2.33 | 8.19 | 28.94 | 23.5 | 0.26 | 0.01 | 3.8 | 1.7 | 0.01 | 0.3 | 0 |
| Bosn3M1 | 0.12 | 1.03 | 10.82 | 31.18 | 17.97 | 0.31 | 0.04 | 3.14 | 1.53 | 0.04 | 0.41 | 0.05 |
| Bosn3M1 | 0.12 | 0.55 | 5.89 | 36.73 | 16.69 | 0.32 | 0.01 | 4.31 | 1.5 | 0.03 | 0.36 | 0 |
| Bosn3M1 | 0.06 | 3.47 | 10.64 | 32.7 | 12.23 | 0.2 | 0.02 | 2.35 | 1.4 | 0 | 0.4 | 0.04 |
| Bosn3M1 | 0.1 | 1.68 | 13.61 | 28.63 | 17.4 | 0.18 | 0 | 2.61 | 1.29 | 0.09 | 0.34 | 0.11 |
| Bosn3M1 | 0.1 | 1.73 | 11.18 | 32.29 | 14.54 | 0.13 | 0.01 | 1.76 | 1.55 | 0 | 0.48 | 0 |
| Bosn3M1 | 0.09 | 0.73 | 13.77 | 30.14 | 16.81 | 0.13 | 0 | 2.08 | 1.63 | 0.01 | 0.35 | 0 |
| Bosn3M1 | 0.1 | 2.93 | 7.49 | 30.53 | 19.71 | 0.37 | 0.02 | 3.81 | 1.87 | 0.01 | 0.34 | 0 |
| Bosn3M1 | 0.12 | 1.23 | 7.72 | 32.83 | 18.09 | 0.34 | 0 | 3.47 | 1.74 | 0.04 | 0.37 | 0.01 |
| Bosn3M1 | 0.12 | 0.61 | 11.64 | 30 | 19.3 | 0.28 | 0.01 | 2.68 | 1.27 | 0.02 | 0.4 | 0.01 |
| Bosn3M1 | 0.15 | 0.58 | 9.53 | 31.22 | 19.27 | 0.31 | 0 | 3.39 | 1.48 | 0.02 | 0.35 | 0 |
| Bosn3M2 | 0.12 | 0.56 | 9.18 | 34.37 | 15.37 | 0.14 | 0.01 | 2.69 | 1.53 | 0.04 | 0.41 | 0 |
| Bosn3M2 | 0.1 | 1.55 | 10.6 | 30.78 | 17.62 | 0.51 | 0.11 | 2.51 | 1.41 | 0.05 | 0.46 | 0.07 |
| Bosn3M2 | 0.08 | 2.53 | 7.98 | 33.14 | 16.53 | 0.43 | 0 | 2.8 | 1.78 | 0 | 0.37 | 0.01 |
| Bosn3M2 | 0.09 | 1.12 | 8.64 | 35.49 | 14.35 | 0.14 | 0 | 1.95 | 1.63 | 0.04 | 0.4 | 0.05 |
| Bosn3M2 | 0.1 | 0.63 | 13.36 | 30.31 | 16.65 | 0.24 | 0.01 | 2.15 | 1.37 | 0 | 0.36 | 0.05 |
| Bosn3M2 | 0.11 | 0.52 | 11.85 | 31.23 | 17.57 | 0.09 | 0 | 2.72 | 1.83 | 0.07 | 0.37 | 0.05 |
| Bosn3M2 | 0.1 | 0.91 | 12.17 | 31.69 | 16.83 | 0.24 | 0.02 | 2.26 | 1.5 | 0.03 | 0.43 | 0.11 |
| Bosn3M2 | 0.11 | 0.44 | 9.63 | 31.5 | 19.8 | 0.22 | 0.02 | 2.97 | 1.67 | 0 | 0.41 | 0.04 |
| Bosn3M2 | 0.13 | 0.42 | 5.89 | 36.6 | 16.58 | 0.15 | 0.02 | 3.16 | 1.8 | 0.07 | 0.3 | 0.01 |
| Bosn3M2 | 0.11 | 0.46 | 11.03 | 30.98 | 18.36 | 0.22 | 0.01 | 1.98 | 1.82 | 0.02 | 0.38 | 0.01 |
| Bosn3M2 | 0.14 | 0.7 | 12.49 | 29.13 | 16.92 | 0.17 | 0.02 | 1.91 | 1.55 | 0.2 | 0.38 | 0.07 |
| Bosn3M2 | 0.08 | 0.82 | 17.96 | 25 | 13.15 | 0.05 | 0 | 1.59 | 1.09 | 0 | 0.34 | 0.01 |
| Bosn3M2 | 0.06 | 0.41 | 10.9 | 32.91 | 12.07 | 0.12 | 0 | 1.64 | 1.13 | 0 | 0.36 | 0.01 |
| Bosn3M2 | 0.14 | 0.75 | 11.02 | 26.88 | 23.91 | 0.24 | 0.02 | 1.8 | 1.72 | 0 | 0.41 | 0 |
| Bosn3M2 | 0.11 | 0.49 | 16.2 | 25.4 | 16.19 | 0.09 | 0.01 | 2.07 | 0.88 | 0.04 | 0.28 | 0 |
| Bosn4M1 | 0.16 | 2.51 | 5.92 | 33.19 | 20.74 | 0.25 | 0.01 | 3.18 | 2.47 | 0.02 | 0.06 | 0 |
| Bosn4M1 | 0.15 | 2.95 | 5.78 | 32.82 | 21.02 | 0.26 | 0.02 | 3.32 | 2.86 | 0.18 | 0.02 | 0.01 |
| Bosn4M1 | 0.14 | 2.07 | 5.55 | 32.8 | 21.89 | 0.45 | 0 | 4.44 | 2.81 | 0 | 0.07 | 0 |
| Bosn4M1 | 0.14 | 2.52 | 4.93 | 32.27 | 22.21 | 0.33 | 0 | 3.05 | 2.69 | 0.08 | 0.07 | 0.01 |
| Bosn4M1 | 0.07 | 3.66 | 7.89 | 30.7 | 17.78 | 0.09 | 0 | 1.89 | 2.44 | 0.02 | 0.11 | 0.01 |
| Bosn4M1 | 0.16 | 2.79 | 5.77 | 31.73 | 20.8 | 0.21 | 0 | 3.41 | 2.59 | 0.09 | 0.06 | 0.11 |
| Bosn4M1 | 0.12 | 4.59 | 8.33 | 29.98 | 18.91 | 0.18 | 0 | 2.05 | 2.6 | 0.06 | 0.11 | 0.05 |
| Bosn4M1 | 0.13 | 1.84 | 5.21 | 34.13 | 19.78 | 0.2 | 0 | 2.71 | 3.28 | 0.09 | 0.15 | 0.01 |
| Bosn4M1 | 0.14 | 2.51 | 6.53 | 31.08 | 21.76 | 0.23 | 0 | 2.97 | 2.73 | 0.03 | 0.03 | 0 |
| Bosn4M1 | 0.12 | 2.68 | 5.19 | 30.89 | 23.86 | 0.18 | 0 | 3.37 | 2.44 | 0.01 | 0.12 | 0 |
| Bosn4M1 | 0.17 | 2.29 | 4.96 | 31.87 | 22.37 | 0.53 | 0 | 2.94 | 2.56 | 0.05 | 0.07 | 0 |
| Bosn4M1 | 0.12 | 3.27 | 6.66 | 30.57 | 20.69 | 0.51 | 0.03 | 2.08 | 2.78 | 0.08 | 0.1 | 0 |
| Bosn4M1 | 0.12 | 2.41 | 6.15 | 31.97 | 21.26 | 0.63 | 0.05 | 3.22 | 2.87 | 0.02 | 0.08 | 0 |
| Bosn4M1 | 0.1 | 2.26 | 4.95 | 32.9 | 22.03 | 0.11 | 0 | 3.06 | 2.48 | 0.04 | 0.12 | 0.04 |
| Bosn4M1 | 0.13 | 3 | 6.14 | 30.94 | 21.17 | 0.24 | 0.02 | 2.48 | 2.87 | 0.06 | 0.07 | 0 |
| Bosn4M2 | 0.13 | 2.86 | 5.35 | 32.75 | 20.65 | 0.17 | 0.03 | 2.5 | 2.57 | 0 | 0.09 | 0.01 |
| Bosn4M2 | 0.16 | 2.04 | 5.56 | 32.35 | 22.77 | 0.42 | 0 | 3.38 | 3.11 | 0.03 | 0.01 | 0.01 |
| Bosn4M2 | 0.14 | 2.85 | 6.71 | 31.81 | 20.7 | 0.42 | 0 | 3.13 | 2.93 | 0.01 | 0.07 | 0 |
| Bosn4M2 | 0.12 | 3.11 | 7.09 | 30.29 | 21.69 | 0.22 | 0.01 | 2.28 | 2.72 | 0.01 | 0.09 | 0.02 |
| Bosn4M2 | 0.13 | 4.49 | 9.25 | 29.72 | 20.45 | 0.13 | 0 | 2.52 | 2.69 | 0.04 | 0.13 | 0.01 |
| Bosn4M2 | 0.13 | 3.67 | 0.54 | 29.74 | 20.88 | 0.49 | 0 | 3.1 | 2.76 | 0.05 | 0.1 | 0 |
| Bosn4M2 | 0.11 | 3.27 | 6.93 | 30.75 | 20.12 | 0.38 | 0.06 | 2.4 | 2.45 | 0.09 | 0.08 | 0 |
| Bosn4M2 | 0.11 | 3.38 | 7.24 | 31.37 | 19.2 | 0.2 | 0 | 2.93 | 2.78 | 0 | 0.1 | 0 |
| Bosn4M2 | 0.17 | 2.52 | 4.45 | 33.27 | 21.9 | 0.19 | 0.01 | 2.07 | 2.86 | 0.01 | 0.1 | 0 |
| Bosn4M2 | 0.1 | 4.64 | 7.9 | 29.39 | 19.64 | 0.23 | 0 | 2.36 | 2.04 | 0.04 | 0.03 | 0 |
| Bosn4M2 | 0.12 | 2.99 | 5.51 | 32.01 | 20.12 | 0.23 | 0 | 3.38 | 2.94 | 0.02 | 0.13 | 0.04 |
| Bosn4M2 | 0.13 | 2.29 | 4.77 | 32.94 | 21.79 | 0.2 | 0 | 2.81 | 2.53 | 0.04 | 0.04 | 0.01 |
| Bosn4M2 | 0.15 | 2.47 | 5.15 | 31.31 | 24.64 | 0.23 | 0.02 | 2.46 | 2.85 | 0 | 0.13 | 0.05 |
| Bosn4M2 | 0.12 | 3.19 | 8.06 | 30.57 | 19.82 | 0.88 | 0.01 | 2.81 | 2.44 | 0.05 | 0.02 | 0.01 |
| Bosn4M2 | 0.12 | 3.37 | 7.53 | 31.42 | 19.16 | 0.21 | 0 | 3.62 | 2.65 | 0.11 | 0.04 | 0 |
| Bosn5M1 | 0.1 | 0.7 | 13.1 | 24.23 | 27.28 | 0.44 | 0.02 | 3.26 | 2 | 0.07 | 0.3 | 0.05 |
| Bosn5M1 | 0.12 | 0.83 | 10.97 | 25.41 | 23.04 | 0.02 | 0.01 | 3.04 | 1.48 | 0 | 0.33 | 0 |
| Bosn5M1 | 0.12 | 1.45 | 18.17 | 21.33 | 23.07 | 0.36 | 0 | 2.72 | 1.83 | 0 | 0.33 | 0.06 |
| Bosn5M1 | 0.1 | 0.64 | 11.69 | 25.77 | 25.17 | 0.22 | 0.03 | 3.89 | 1.51 | 0 | 0.37 | 0 |
| Bosn5M1 | 0.15 | 0.84 | 12.65 | 20.21 | 32.54 | 0.18 | 0 | 4.77 | 1.81 | 0.03 | 0.28 | 0 |
| Bosn5M1 | 0.05 | 1.07 | 21.75 | 19.33 | 20.34 | 0.02 | 0.02 | 2.6 | 0.91 | 0.05 | 0.24 | 0.05 |
| Bosn5M1 | 0.11 | 0.68 | 12.28 | 27.75 | 21.41 | 0.07 | 0.04 | 2.89 | 1.43 | 0 | 0.32 | 0 |
| Bosn5M1 | 0.15 | 0.62 | 16.65 | 20.31 | 25.27 | 0 | 0.01 | 3.66 | 1.04 | 0 | 0.3 | 0 |
| Bosn5M1 | 0.08 | 0.36 | 10.33 | 28.9 | 22.65 | 0.13 | 0.01 | 3.24 | 1.42 | 0.06 | 0.33 | 0.03 |
| Bosn5M1 | 0.14 | 0.56 | 10.22 | 21.9 | 33.37 | 0.08 | 0 | 4.76 | 1.75 | 0 | 0.27 | 0.05 |
| Bosn5M1 | 0.11 | 1.13 | 16.2 | 22.8 | 19.66 | 0.01 | 0 | 2.75 | 1.32 | 0.02 | 0.34 | 0.09 |
| Bosn5M1 | 0.11 | 0.95 | 20.07 | 19.14 | 21.72 | 0 | 0 | 2.33 | 1.1 | 0 | 0.23 | 0 |
| Bosn5M1 | 0.14 | 0.75 | 19.37 | 20.23 | 22.88 | 0 | 0.01 | 2.49 | 1.08 | 0.03 | 0.31 | 0 |
| Bosn5M1 | 0.1 | 1.02 | 20.15 | 19.14 | 26.71 | 0.03 | 0 | 2.22 | 1.11 | 0 | 0.32 | 0 |
| Bosn5M1 | 0.11 | 1.68 | 14.8 | 22.49 | 26.08 | 0.13 | 0.01 | 4.87 | 1.38 | 0.03 | 0.28 | 0 |
| Bosn5M2 | 0.1 | 1.23 | 15.07 | 24.14 | 22.45 | 0.09 | 0.01 | 2.54 | 1.49 | 0 | 0.36 | 0 |
| Bosn5M2 | 0.12 | 2.03 | 13.91 | 24.68 | 20.01 | 0.24 | 0.03 | 2.17 | 2.06 | 0.03 | 0.4 | 0.04 |
| Bosn5M2 | 0.1 | 2.27 | 16.72 | 27.07 | 15.53 | 0.17 | 0 | 1.55 | 1.51 | 0 | 0.38 | 0 |
| Bosn5M2 | 0.1 | 0.79 | 9.46 | 31.74 | 17.16 | 0.25 | 0.03 | 2.03 | 1.76 | 0.03 | 0.45 | 0 |
| Bosn5M2 | 0.12 | 1.09 | 13.66 | 29.16 | 17.84 | 0.08 | 0.04 | 2.06 | 1.57 | 0.12 | 0.31 | 0.01 |
| Bosn5M2 | 0.09 | 1.68 | 16.89 | 25.24 | 19.53 | 0 | 0.01 | 1.75 | 1.42 | 0 | 0.36 | 0 |
| Bosn5M2 | 0.14 | 0.74 | 8.36 | 28.89 | 23.96 | 0.12 | 0.01 | 3.5 | 1.79 | 0.04 | 0.4 | 0.03 |
| Bosn5M2 | 0.18 | 0.94 | 5.44 | 33.02 | 22.97 | 0.62 | 0.03 | 4.84 | 2.51 | 0.04 | 0.52 | 0.02 |
| Bosn5M2 | 0.1 | 0.95 | 17.59 | 24.26 | 19.6 | 0 | 0.03 | 2.93 | 1.12 | 0.01 | 0.35 | 0 |
| Bosn5M2 | 0.11 | 0.68 | 12.57 | 24.87 | 24.77 | 0.26 | 0.05 | 3.92 | 1.36 | 0.09 | 0.4 | 0 |
| Bosn5M2 | 0.14 | 0.64 | 12.19 | 25.23 | 23.36 | 0.06 | 0.01 | 3.52 | 1.25 | 0.01 | 0.37 | 0 |
| Bosn5M2 | 0.1 | 0.89 | 14.09 | 26.68 | 19.7 | 0.03 | 0.01 | 2.48 | 1.21 | 0 | 0.27 | 0.04 |
| Bosn5M2 | 0.11 | 2.18 | 16.27 | 20.36 | 24.7 | 0.05 | 0.02 | 3.53 | 1.81 | 0.06 | 0.24 | 0.01 |
| Bosn5M2 | 0.16 | 0.75 | 17.17 | 18.62 | 32.26 | 0 | 0 | 2 | 0.85 | 0.05 | 0.21 | 0.05 |
| Bosn5M2 | 0.08 | 1.18 | 12.77 | 23.44 | 27.45 | 0.19 | 0.01 | 3.22 | 1.76 | 0 | 0.41 | 0 |
| Bosn6M1 | 0.24 | 0.62 | 10.06 | 29.68 | 22.16 | 0.32 | 0.03 | 4.36 | 2.06 | 0.07 | 0.51 | 0 |
| Bosn6M1 | 0.14 | 1.38 | 18.81 | 25.55 | 16.64 | 0.12 | 0.03 | 2.78 | 1.52 | 0.04 | 0.27 | 0.04 |
| Bosn6M1 | 0.2 | 0.52 | 11 | 32.31 | 19.1 | 0.08 | 0 | 2.48 | 2.13 | 0 | 0.44 | 0.05 |
| Bosn6M1 | 0.22 | 1 | 15.58 | 28.17 | 19.44 | 0.04 | 0.01 | 3.73 | 1.7 | 0.09 | 0.52 | 0.02 |
| Bosn6M1 | 0.25 | 0.65 | 18.6 | 24.72 | 17.45 | 0 | 0.01 | 2.3 | 1.34 | 0.06 | 0.3 | 0.07 |
| Bosn6M1 | 0.3 | 0.96 | 15.74 | 25.37 | 16.82 | 0.16 | 0.01 | 3.5 | 1.6 | 0.06 | 0.43 | 0 |
| Bosn6M1 | 0.23 | 0.7 | 20.62 | 20.57 | 15.76 | 0 | 0 | 2.91 | 0.96 | 0.06 | 0.32 | 0 |
| Bosn6M1 | 0.19 | 0.88 | 19.59 | 26.34 | 16.57 | 0.05 | 0.02 | 2.78 | 1.02 | 0 | 0.43 | 0 |
| Bosn6M1 | 0.19 | 0.61 | 17.59 | 25.62 | 18.15 | 0.06 | 0.02 | 2.56 | 1.29 | 0.06 | 0.43 | 0.06 |
| Bosn6M1 | 0.23 | 0.67 | 13.56 | 25.49 | 21.2 | 0.22 | 0 | 3.17 | 1.84 | 0.01 | 0.34 | 0 |
| Bosn6M1 | 0.22 | 1.05 | 17.55 | 25 | 19.45 | 0.03 | 0.02 | 3.03 | 1.57 | 0.01 | 0.38 | 0 |
| Bosn6M1 | 0.18 | 0.84 | 19.17 | 24.42 | 14.82 | 0.02 | 0 | 2.59 | 1.6 | 0.02 | 0.38 | 0.02 |
| Bosn6M1 | 0.24 | 0.69 | 15.39 | 24.99 | 18.82 | 0.09 | 0.01 | 3.37 | 1.61 | 0 | 0.42 | 0.01 |
| Bosn6M1 | 0.21 | 0.75 | 16.86 | 23.3 | 17.23 | 0.15 | 0 | 2.55 | 1.48 | 0 | 0.31 | 0 |
| Bosn6M1 | 0.19 | 0.58 | 16.38 | 26.8 | 17.22 | 0.27 | 0.01 | 3.57 | 1.46 | 0.01 | 0.38 | 0 |
| Bosn6M2 | 0.24 | 0.87 | 16.77 | 24 | 20.05 | 0.21 | 0 | 3.47 | 1.88 | 0 | 0.38 | 0 |
| Bosn6M2 | 0.23 | 0.65 | 18.73 | 25.71 | 18 | 0.21 | 0 | 2.02 | 1.44 | 0.03 | 0.34 | 0 |
| Bosn6M2 | 0.19 | 0.86 | 19.98 | 26.81 | 14.04 | 0 | 0.02 | 2.36 | 1.2 | 0.02 | 0.4 | 0.03 |
| Bosn6M2 | 0.2 | 0.6 | 13.15 | 28.15 | 18.11 | 0.51 | 0.1 | 4.22 | 2.61 | 0.06 | 0.4 | 0.01 |
| Bosn6M2 | 0.22 | 0.56 | 9.45 | 30.1 | 23.32 | 0.25 | 0.01 | 4.9 | 1.91 | 0.02 | 0.58 | 0.05 |
| Bosn6M2 | 0.21 | 1.25 | 13.47 | 26.5 | 21.71 | 0.08 | 0.01 | 2.47 | 1.52 | 0 | 0.51 | 0 |
| Bosn6M2 | 0.23 | 0.75 | 14.22 | 26.87 | 20.63 | 0.08 | 0 | 2.67 | 1.6 | 0.02 | 0.36 | 0 |
| Bosn6M2 | 0.26 | 0.59 | 9.51 | 29.5 | 25.41 | 0.3 | 0.01 | 6.19 | 2.21 | 0.02 | 0.41 | 0 |
| Bosn6M2 | 0.21 | 0.67 | 13.08 | 29.39 | 20.07 | 0.15 | 0.01 | 3.97 | 1.69 | 0 | 0.42 | 0.02 |
| Bosn6M2 | 0.2 | 1.42 | 16.99 | 25.6 | 18.58 | 0.23 | 0.04 | 2.87 | 1.42 | 0.05 | 0.49 | 0 |
| Bosn6M2 | 0.23 | 0.92 | 14.5 | 26.17 | 20.95 | 0.08 | 0 | 4.55 | 1.43 | 0.03 | 0.4 | 0.02 |
| Bosn6M2 | 0.19 | 1.04 | 20.11 | 26.26 | 15.98 | 0.04 | 0.05 | 2.75 | 1.47 | 0.04 | 0.41 | 0.03 |
| Bosn6M2 | 0.21 | 0.53 | 6.57 | 32.8 | 21.22 | 0.28 | 0.02 | 5.41 | 2.16 | 0 | 0.49 | 0 |
| Bosn6M2 | 0.2 | 1.89 | 12.71 | 26.4 | 22.54 | 0.07 | 0.01 | 2.91 | 1.6 | 0.04 | 0.39 | 0.11 |
| Bosn6M2 | 0.23 | 1.1 | 12.82 | 28.49 | 19.59 | 0.24 | 0 | 4.23 | 2.01 | 0.05 | 0.48 | 0 |
| ChinaM1 | 0.17 | 1.872 | 17.353 | 31.103 | 6.851 | 0.751 | 0.292 | 2.062 | 2.176 | 0.161 | 0.405 | 0.049 |
| ChinaM1 | 0.149 | 1.441 | 8.949 | 38.532 | 8.839 | 0.267 | 0.045 | 2.806 | 2.945 | 0 | 0.767 | 0.008 |
| ChinaM1 | 0.183 | 0.792 | 9.122 | 38.62 | 8.771 | 0.283 | 0.017 | 3.158 | 2.507 | 0.062 | 0.946 | 0 |
| ChinaM1 | 0.183 | 0.75 | 8.596 | 38.902 | 8.839 | 0.233 | 0.041 | 2.909 | 3.623 | 0.037 | 0.9 | 0 |
| ChinaM1 | 0.165 | 2.319 | 12.294 | 33.01 | 8.541 | 1.864 | 0.331 | 2.5 | 4.576 | 1.682 | 0.748 | 0.072 |
| ChinaM1 | 0.175 | 1.119 | 9.801 | 37.976 | 8.426 | 0.102 | 0 | 3.357 | 2.761 | 0 | 0.814 | 0.057 |
| ChinaM1 | 0.133 | 1.548 | 12.158 | 35.053 | 8.373 | 0.205 | 0.051 | 3.361 | 3.165 | 0.011 | 0.754 | 0.057 |
| ChinaM1 | 0.114 | 1.962 | 12.415 | 34.807 | 7.505 | 0.534 | 0.175 | 2.743 | 2.423 | 0.436 | 0.617 | 0 |
| ChinaM1 | 0.094 | 2.295 | 15.929 | 32.523 | 6.766 | 0.142 | 0.05 | 2.766 | 3.849 | 0.011 | 0.539 | 0.089 |
| ChinaM1 | 0.143 | 2.632 | 13.984 | 32.991 | 6.889 | 1.497 | 0.136 | 2.631 | 2.555 | 0.084 | 0.539 | 0.178 |
| ChinaM1 | 0.194 | 1.244 | 13.603 | 34.968 | 7.597 | 0.106 | 0.017 | 2.32 | 1.629 | 0.059 | 0.706 | 0.049 |
| ChinaM1 | 0.156 | 1.325 | 10.754 | 36.771 | 7.688 | 0.479 | 0.216 | 2.201 | 2.118 | 0.531 | 0.874 | 0 |
| ChinaM1 | 0.217 | 1.176 | 10.874 | 37.178 | 7.618 | 0.067 | 0.002 | 2.62 | 1.553 | 0.04 | 0.856 | 0.065 |
| ChinaM1 | 0.203 | 1.635 | 12.407 | 32.907 | 7.088 | 0.019 | 0 | 1.86 | 1.259 | 0.004 | 0.726 | 0 |
| ChinaM1 | 0.236 | 1.411 | 13.93 | 34.59 | 7.82 | 0.062 | 0.008 | 2.116 | 1.556 | 0.044 | 0.8 | 0 |
| ChinaM2 | 0.227 | 1.134 | 11.807 | 36.794 | 8.607 | 0.508 | 0.081 | 2.843 | 3.697 | 0.598 | 0.809 | 0 |
| ChinaM2 | 0.188 | 1.016 | 10.782 | 38.335 | 8.809 | 0.492 | 0.077 | 3.098 | 3.964 | 0.117 | 0.864 | 0.04 |
| ChinaM2 | 0.157 | 1.751 | 11.295 | 36.81 | 8.384 | 0.916 | 0.178 | 2.885 | 3.92 | 0.29 | 0.793 | 0.032 |
| ChinaM2 | 0.206 | 1.678 | 9.708 | 38.352 | 8.629 | 0.82 | 0.078 | 2.887 | 3.467 | 0.355 | 0.804 | 0.137 |
| ChinaM2 | 0.162 | 1.057 | 9.652 | 38.691 | 8.686 | 0.226 | 0.024 | 2.632 | 2.773 | 0 | 0.931 | 0.04 |
| ChinaM2 | 0.159 | 3.164 | 9.19 | 36.326 | 7.936 | 1.899 | 0.225 | 2.573 | 4.177 | 0.575 | 0.773 | 0 |
| ChinaM2 | 0.203 | 3.623 | 11.567 | 34.805 | 7.49 | 0.467 | 0.051 | 2.471 | 3.09 | 0.033 | 0.754 | 0 |
| ChinaM2 | 0.152 | 1.286 | 6.774 | 36.463 | 8.679 | 7.412 | 0.547 | 2.643 | 4.687 | 0.641 | 0.51 | 0.145 |
| ChinaM2 | 0.181 | 3.429 | 8.144 | 36.633 | 7.357 | 2.407 | 0.379 | 2.599 | 4.587 | 0.367 | 0.618 | 0.113 |
| ChinaM2 | 0.165 | 2.006 | 14.527 | 33.867 | 7.337 | 0.216 | 0.054 | 2.736 | 4.122 | 0.029 | 0.664 | 0.073 |
| ChinaM2 | 0.197 | 2.573 | 12.56 | 32.411 | 8.054 | 2.853 | 0.472 | 2.655 | 3.514 | 0.447 | 0.593 | 0.008 |
| ChinaM2 | 0.186 | 1.819 | 10.617 | 36.381 | 8.488 | 0.213 | 0.021 | 3.123 | 2.65 | 0.011 | 0.705 | 0.008 |
| ChinaM2 | 0.237 | 0.918 | 6.413 | 40.396 | 9.372 | 0.129 | 0.005 | 3.727 | 2.53 | 0 | 0.792 | 0.032 |
| ChinaM2 | 0.185 | 1.064 | 6.082 | 40.362 | 9.468 | 0.184 | 0 | 3.312 | 2.152 | 0.015 | 0.616 | 0.065 |
| ChinaM2 | 0.12 | 1.025 | 6.643 | 39.09 | 8.287 | 4.317 | 0.743 | 3.756 | 5.823 | 0.445 | 0.662 | 0.081 |
| GerMaple | 0.46 | 7.257 | 21.37 | 24.547 | 5.997 | 0.418 | 0.542 | 4.154 | 0.684 | 0 | 2.709 | 0.035 |
| GerMaple | 0.447 | 3.075 | 30.935 | 20.561 | 3.1 | 0.778 | 0.309 | 2.529 | 1.945 | 0.169 | 2.986 | 0 |
| GerMaple | 0.557 | 3.782 | 29.926 | 20.773 | 4.308 | 0.568 | 0.4 | 1.778 | 0.863 | 0.047 | 1.707 | 0.07 |
| GerMaple | 0.523 | 2.292 | 30.102 | 18.534 | 5.754 | 2.761 | 0.182 | 1.126 | 0.902 | 0.044 | 2.479 | 0 |
| GerMaple | 0.432 | 1.364 | 31.611 | 17.58 | 5.795 | 0.832 | 0.099 | 1.138 | 0.496 | 0 | 2.729 | 0 |
| GerMaple | 0.63 | 2.693 | 30.812 | 17.526 | 5.447 | 0.567 | 0.301 | 2.071 | 0.722 | 0 | 2.414 | 0 |
| GerMaple | 0.602 | 1.946 | 29.467 | 18.144 | 5.827 | 1.05 | 0.659 | 1.424 | 1.084 | 0.052 | 3.007 | 0.005 |
| GerMaple | 0.602 | 1.035 | 32.259 | 17.169 | 4.474 | 0.743 | 0.525 | 1.611 | 0.471 | 0 | 2.052 | 0 |
| GerMaple | 0.509 | 0.937 | 32.029 | 20.521 | 3.219 | 0.676 | 0.371 | 1.883 | 1.211 | 0.04 | 1.575 | 0.065 |
| GerMaple | 0.471 | 1.153 | 32.201 | 18.451 | 4.84 | 0.752 | 0.427 | 0.823 | 1.042 | 0 | 2.416 | 0.1 |
| GerMaple | 0.313 | 0.49 | 31.197 | 20.837 | 5.582 | 0.797 | 0.216 | 0.683 | 0.365 | 0 | 2.793 | 0 |
| GerMaple | 0.207 | 0.588 | 22.534 | 32.803 | 3.564 | 0.285 | 0.111 | 0.475 | 0.303 | 0 | 1.986 | 0.035 |
| GerMaple | 1.28 | 1.617 | 33.263 | 16.766 | 4.174 | 0.517 | 0.24 | 0.546 | 0.386 | 0 | 1.799 | 0 |
| GerMaple | 0.719 | 1.125 | 23.935 | 29.383 | 3.233 | 0.975 | 0.361 | 0.373 | 0.73 | 0.037 | 2.369 | 0 |
| GerMaple | 0.644 | 1.335 | 32.656 | 17.907 | 4.371 | 0.688 | 0.141 | 0.654 | 0.437 | 0 | 2.367 | 0 |
| slven1-ht | 0.23 | 1.36 | 25.07 | 28.32 | 4.33 | 1.33 | 0.26 | 2.16 | 1.74 | 0.1 | 0.08 | 0.05 |
| slven1-ht | 0.23 | 0.85 | 21.93 | 30.86 | 5.2 | 1.23 | 0.21 | 3.24 | 2.18 | 0.07 | 0.17 | 0.05 |
| slven1-ht | 0.34 | 1.97 | 13.99 | 33.64 | 5.18 | 8.19 | 2.58 | 3.1 | 3.07 | 0.61 | 0.12 | 0.25 |
| slven1-ht | 0.25 | 2.45 | 22.5 | 28.36 | 3.97 | 4.38 | 1.32 | 2.17 | 2.58 | 0.47 | 0.09 | 0.28 |
| slven1-ht | 0.17 | 3.52 | 20.87 | 28.45 | 4.01 | 5.21 | 1.71 | 1.79 | 2.32 | 0.51 | 0.17 | 0.01 |
| slven1-ht | 0.13 | 2.94 | 22.38 | 30.1 | 4.37 | 0.64 | 0.08 | 1.86 | 2.17 | 0.05 | 0.16 | 0 |
| slven1-ht | 0.23 | 1.89 | 24.3 | 27.24 | 4.59 | 3.61 | 1.23 | 2.27 | 1.92 | 0.48 | 0.13 | 0.21 |
| slven1-ht | 0.2 | 3.99 | 18.62 | 31.07 | 6.65 | 0.69 | 0.01 | 4.29 | 2.11 | 0.03 | 0.2 | 0.02 |
| slven1-ht | 0.19 | 2.89 | 18.88 | 32.22 | 6.51 | 0.69 | 0.07 | 3.04 | 2.16 | 0.3 | 0.09 | 0.01 |
| slven1-ht | 0.22 | 0.83 | 24.57 | 31.32 | 5.61 | 0.86 | 0.19 | 2.44 | 2.29 | 0.12 | 0.12 | 0 |
| slven1-ht | 0.25 | 0.64 | 22.71 | 31.98 | 6.41 | 0.19 | 0 | 3.14 | 2.02 | 0.01 | 0.15 | 0.08 |
| slven1-ht | 0.16 | 0.57 | 19.05 | 34.34 | 6.27 | 0.76 | 0.02 | 4.23 | 2.26 | 0.04 | 0.1 | 0 |
| slven1-ht | 0.2 | 3.14 | 27 | 24.63 | 3.41 | 3.6 | 0.59 | 1.72 | 1.84 | 0.12 | 0.22 | 0.06 |
| slven1-ht | 0.22 | 3.34 | 16.12 | 27.91 | 4.9 | 9.74 | 3.96 | 3.09 | 2.46 | 0.23 | 0.16 | 0.09 |
| slven1-ht | 0.2 | 3.53 | 23.39 | 28.31 | 4.72 | 0.54 | 0.01 | 2.7 | 1.91 | 0.05 | 0.13 | 0 |
| slven2-ht | 0.21 | 1.07 | 18.95 | 34.18 | 6.81 | 0.66 | 0.1 | 3.89 | 3.04 | 0 | 0.16 | 0 |
| slven2-ht | 0.41 | 2.27 | 23.54 | 30.13 | 4.91 | 0.56 | 0.04 | 2.28 | 2.54 | 0.01 | 0.16 | 0 |
| slven2-ht | 0.25 | 4.31 | 20.52 | 26.91 | 4.08 | 6.93 | 2.58 | 1.95 | 2.68 | 0.69 | 0.11 | 0.12 |
| slven2-ht | 0.54 | 3.67 | 24.31 | 27.35 | 4.01 | 2.96 | 0.91 | 1.88 | 2.31 | 0.17 | 0.08 | 0.03 |
| slven2-ht | 0.19 | 1.84 | 24.09 | 29.16 | 5.56 | 1.64 | 0.49 | 3.05 | 2.32 | 0.2 | 0.2 | 0.14 |
| slven2-ht | 0.17 | 3.47 | 21.87 | 28.17 | 6.32 | 3.47 | 1.09 | 2.97 | 3.22 | 0.29 | 0.13 | 0 |
| slven2-ht | 0.23 | 2.79 | 23.34 | 29.48 | 6.31 | 0.44 | 0 | 2.97 | 2.36 | 0 | 0.13 | 0.12 |
| slven2-ht | 0.23 | 2.06 | 23.34 | 26.64 | 4.2 | 7.07 | 1.65 | 2.18 | 2.24 | 0.49 | 0.08 | 0.88 |
| slven2-ht | 0.18 | 5.08 | 23.06 | 26.57 | 4.24 | 2.12 | 0.63 | 1.65 | 1.81 | 0.22 | 0.19 | 0.07 |
| slven2-ht | 0.27 | 2.23 | 25.05 | 25.99 | 4.07 | 5.16 | 2.04 | 2.06 | 2.05 | 0.52 | 0.13 | 0.01 |
| slven2-ht | 0.26 | 0.93 | 25.65 | 28.77 | 5.09 | 0.52 | 0.03 | 2.11 | 2.12 | 0 | 0.12 | 0.03 |
| slven2-ht | 0.54 | 2.58 | 15.25 | 26.61 | 4.1 | 14.98 | 5.25 | 2.45 | 4.11 | 1.54 | 0.17 | 0.14 |
| slven2-ht | 0.24 | 2.57 | 22.35 | 28.27 | 6.1 | 0.45 | 0.01 | 3.4 | 2.12 | 0 | 0.1 | 0.04 |
| slven2-ht | 0.25 | 1.42 | 21.76 | 29.84 | 5.53 | 2.61 | 0.68 | 3.08 | 2.39 | 0.14 | 0.12 | 0.09 |
| slven2-ht | 0.23 | 2.27 | 25.14 | 27.16 | 4.36 | 1.42 | 0.35 | 2.02 | 2.01 | 0.04 | 0.09 | 0.05 |
| slven1-sp | 0.58 | 0.67 | 12 | 37.7 | 9.55 | 0.78 | 0.02 | 9.88 | 4.71 | 0.01 | 0.19 | 0.05 |
| slven1-sp | 0.52 | 4.84 | 18.12 | 29.41 | 5.58 | 0.7 | 0.02 | 7.39 | 3.3 | 0.01 | 0.2 | 0 |
| slven1-sp | 0.62 | 0.68 | 27.32 | 26.15 | 5.22 | 0.69 | 0.03 | 7.04 | 2.6 | 0.05 | 0.2 | 0 |
| slven1-sp | 0.41 | 4.39 | 21.64 | 27.88 | 5.3 | 0.59 | 0.01 | 4.28 | 2.91 | 0.07 | 0.22 | 0 |
| slven1-sp | 1.19 | 2.8 | 16.58 | 32.98 | 7.43 | 0.69 | 0 | 5.26 | 3.74 | 0.03 | 0.2 | 0 |
| slven1-sp | 0.72 | 4.76 | 19.54 | 29.84 | 5.97 | 0.77 | 0 | 5.86 | 3.63 | 0.02 | 0.18 | 0.03 |
| slven1-sp | 0.47 | 6.97 | 15.06 | 30.34 | 6.22 | 0.84 | 0.01 | 6.05 | 4.11 | 0.06 | 0.15 | 0 |
| slven1-sp | 0.5 | 3.71 | 22.15 | 28.39 | 6.11 | 0.54 | 0.03 | 6.74 | 2.92 | 0.13 | 0.18 | 0.05 |
| slven1-sp | 0.36 | 1.38 | 22.97 | 29.74 | 5.89 | 0.47 | 0 | 4.13 | 3.16 | 0.01 | 0.2 | 0 |
| slven1-sp | 0.66 | 4.14 | 19.3 | 28.87 | 7.29 | 1.14 | 0.01 | 7 | 2.79 | 0.06 | 0.13 | 0 |
| slven1-sp | 0.22 | 3.57 | 15.49 | 32.84 | 6.5 | 1.61 | 0.02 | 8.88 | 4.22 | 0.09 | 0.23 | 0.05 |
| slven1-sp | 0.44 | 5.12 | 11.51 | 34.96 | 7.01 | 1.34 | 0.01 | 6.72 | 3.75 | 0.08 | 0.27 | 0.05 |
| slven1-sp | 1.01 | 5.23 | 28.27 | 21.7 | 4.4 | 0.44 | 0 | 4.76 | 1.53 | 0.14 | 0.1 | 0 |
| slven1-sp | 0.35 | 1.38 | 21.41 | 29.47 | 7.26 | 0.7 | 0 | 7.41 | 3.44 | 0.05 | 0.14 | 0.05 |
| slven1-sp | 0.65 | 1.03 | 25.01 | 28.32 | 5.71 | 1 | 0.08 | 3.89 | 3.17 | 0.03 | 0.21 | 0 |
| slven2-sp | 0.37 | 2.91 | 15.39 | 34.14 | 6.33 | 1.33 | 0.02 | 7.23 | 3.9 | 0.12 | 0.26 | 0.01 |
| slven2-sp | 0.49 | 5.66 | 19.41 | 27.91 | 5.82 | 1.45 | 0 | 8.79 | 3.74 | 0.08 | 0.2 | 0.02 |
| slven2-sp | 0.72 | 3.5 | 16.8 | 29.45 | 5.52 | 6.97 | 1.6 | 5.99 | 4.37 | 0.67 | 0.19 | 0.27 |
| slven2-sp | 1.1 | 3.22 | 25.71 | 24.1 | 4.9 | 1.56 | 0.32 | 6.21 | 3.75 | 0.21 | 0.18 | 0.08 |
| slven2-sp | 0.51 | 2.71 | 26.12 | 24.01 | 4.6 | 0.8 | 0.02 | 4.55 | 4.02 | 0.05 | 0.19 | 0.03 |
| slven2-sp | 0.55 | 4.58 | 21.41 | 26.73 | 5.71 | 1.42 | 0.07 | 5.55 | 3.89 | 0 | 0.24 | 0.04 |
| slven2-sp | 0.7 | 5.26 | 20.06 | 27.6 | 5.39 | 1.74 | 0.07 | 8.53 | 3.58 | 0.2 | 0.17 | 0 |
| slven2-sp | 0.58 | 6.65 | 23.56 | 22.57 | 4.8 | 0.7 | 0.03 | 3.3 | 2.15 | 0.15 | 0.16 | 0.06 |
| slven2-sp | 0.54 | 2.89 | 24.82 | 26.44 | 5.16 | 0.81 | 0.01 | 6.3 | 2.74 | 0.03 | 0.16 | 0 |
| slven2-sp | 0.37 | 6.52 | 18.75 | 26.67 | 5.49 | 0.99 | 0.08 | 4.83 | 3.41 | 0.26 | 0.25 | 0.03 |
| slven2-sp | 10.34 | 1.6 | 39.88 | 13.46 | 3 | 0.68 | 0.11 | 2.91 | 3.12 | 0.08 | 0.11 | 0 |
| slven2-sp | 0.26 | 0.99 | 18.33 | 33.18 | 7.62 | 1.02 | 0 | 8.52 | 3.71 | 0.03 | 0.19 | 0 |
| slven2-sp | 1.04 | 1.88 | 21.77 | 26.1 | 5.61 | 1.84 | 0.44 | 9.53 | 4.34 | 0.09 | 0.25 | 0 |
| slven2-sp | 3.08 | 3.21 | 27.97 | 20.43 | 4.88 | 4.37 | 1.09 | 5.14 | 5.29 | 0 | 0.1 | 0.09 |
| slven2-sp | 0.81 | 1.54 | 28.74 | 23.61 | 4.99 | 1.1 | 0.05 | 3.36 | 3.2 | 0.08 | 0.19 | 0 |
| slvk1-ht | 0.611 | 1.128 | 22.293 | 27.412 | 10.918 | 0 | 0 | 2.863 | 1.543 | 0.02 | 0.037 | 0 |
| slvk1-ht | 1.903 | 6.017 | 16.51 | 29.998 | 7.811 | 0.215 | 0.033 | 2.074 | 1.885 | 0.024 | 0.175 | 0.053 |
| slvk1-ht | 0.685 | 0.71 | 3.94 | 42.449 | 11.268 | 0.456 | 0.073 | 3.208 | 2.229 | 0.14 | 0.188 | 0.044 |
| slvk1-ht | 0.692 | 0.682 | 2.759 | 44.91 | 9.176 | 0.294 | 0.005 | 2.162 | 4.3 | 0.303 | 0.111 | 0.044 |
| slvk1-ht | 0.651 | 5.365 | 13.658 | 31.972 | 8.547 | 0.349 | 0.067 | 1.941 | 2.206 | 0.083 | 0.179 | 0 |
| slvk1-ht | 0.736 | 7.471 | 12.843 | 30.483 | 8.716 | 0.109 | 0.017 | 2.398 | 1.976 | 0.175 | 0.029 | 0 |
| slvk1-ht | 0.499 | 3.626 | 12.423 | 32.565 | 9.849 | 0.298 | 0.069 | 2.647 | 2.263 | 0.238 | 0.118 | 0.07 |
| slvk1-ht | 1.895 | 5.057 | 13.908 | 32.093 | 6.352 | 0.427 | 0.084 | 1.77 | 2.187 | 0.134 | 0.093 | 0.079 |
| slvk1-ht | 0.413 | 4.385 | 12.191 | 33.314 | 7.704 | 0.809 | 0.099 | 2.156 | 2.268 | 0.683 | 0.142 | 0.026 |
| slvk1-ht | 1.187 | 2 | 15.181 | 28.788 | 11.476 | 0.339 | 0.009 | 2.371 | 2.066 | 0.412 | 0.138 | 0 |
| slvk1-ht | 2.222 | 5.358 | 10.659 | 36.275 | 5.483 | 0.14 | 0.019 | 1.31 | 2.071 | 0.051 | 0.195 | 0.079 |
| slvk1-ht | 1.527 | 2.379 | 14.424 | 33.56 | 7.686 | 0.118 | 0.027 | 1.681 | 2.28 | 0.17 | 0.171 | 0 |
| slvk1-ht | 0.986 | 1.38 | 6.817 | 41.029 | 8.298 | 0.538 | 0.049 | 1.67 | 2.054 | 0.231 | 0.18 | 0 |
| slvk1-ht | 0.902 | 0.35 | 2.32 | 47.283 | 7.353 | 0.318 | 0.002 | 1.487 | 2.215 | 0.04 | 0.127 | 0 |
| slvk1-ht | 0.61 | 1.292 | 9.181 | 33.941 | 9.973 | 0.14 | 0.006 | 2.541 | 2.48 | 0.207 | 0.126 | 0.053 |
| slvk2-ht | 1.563 | 0.397 | 5.097 | 36.819 | 15.035 | 4.156 | 0.14 | 3.66 | 4.493 | 4.518 | 0.102 | 0 |
| slvk2-ht | 0.731 | 0.776 | 4.671 | 36.566 | 12.357 | 8.562 | 1.82 | 2.892 | 3.408 | 1.051 | 0.291 | 0.211 |
| slvk2-ht | 2.052 | 0.617 | 6.345 | 36.494 | 17.03 | 1.789 | 0.141 | 3.352 | 3.037 | 0.849 | 0.139 | 0.07 |
| slvk2-ht | 1.109 | 0.699 | 6.605 | 38.376 | 14.378 | 0.555 | 0.144 | 3.362 | 4.003 | 0.303 | 0.168 | 0.026 |
| slvk2-ht | 1.993 | 4.601 | 13.755 | 31.86 | 10.638 | 0.442 | 0.024 | 3.602 | 2.201 | 0.111 | 0.346 | 0 |
| slvk2-ht | 2.975 | 1.122 | 9.381 | 38.292 | 9.838 | 0.967 | 0.041 | 2.499 | 3.096 | 0.626 | 0.134 | 0.097 |
| slvk2-ht | 1.732 | 0.35 | 6.401 | 38.194 | 14.106 | 0.469 | 0.128 | 3.726 | 3.466 | 0.303 | 0.176 | 0.132 |
| slvk2-ht | 1.497 | 5.17 | 19.74 | 26.237 | 10.866 | 0 | 0.016 | 2.68 | 1.974 | 0.38 | 0.13 | 0 |
| slvk2-ht | 3.322 | 2.224 | 16.416 | 32.18 | 9.313 | 0 | 0.023 | 2.498 | 2.121 | 0.15 | 0.061 | 0.07 |
| slvk2-ht | 2.598 | 4.746 | 14.82 | 32.062 | 8.876 | 0.028 | 0 | 2.235 | 2.81 | 0 | 0.142 | 0.062 |
| slvk2-ht | 2.844 | 2.39 | 9.637 | 36.7 | 11.447 | 0.205 | 0 | 2.077 | 2.61 | 0.072 | 0.139 | 0.035 |
| slvk2-ht | 1.93 | 0.649 | 3.612 | 41.478 | 13.446 | 0.159 | 0.005 | 3.743 | 2.324 | 0.04 | 0.282 | 0.141 |
| slvk2-ht | 1.728 | 1.183 | 12.889 | 35.522 | 10.026 | 0.467 | 0.022 | 2.487 | 2.925 | 0.135 | 0.212 | 0.009 |
| slvk2-ht | 2.631 | 1.7 | 12.534 | 36.723 | 7.917 | 0.111 | 0 | 2.287 | 3.168 | 0.095 | 0.077 | 0 |
| slvk2-ht | 1.238 | 0.794 | 4.757 | 40.847 | 12.611 | 0.355 | 0.004 | 3.014 | 2.964 | 0.068 | 0.205 | 0.044 |
| slvk1-sp | 0.207 | 0.294 | 25.484 | 18.61 | 6.219 | 2.804 | 0.35 | 6.196 | 2.205 | 1.031 | 0.061 | 0.21 |
| slvk1-sp | 0.282 | 0.457 | 26.856 | 17.615 | 7.427 | 0.148 | 0.002 | 8.953 | 1.84 | 0 | 0.053 | 0.044 |
| slvk1-sp | 0.696 | 1.498 | 27.882 | 19.483 | 5.827 | 0.155 | 0.012 | 5.615 | 1.972 | 0 | 0.097 | 0.079 |
| slvk1-sp | 0.222 | 0.476 | 24.421 | 20.507 | 8.421 | 0.272 | 0 | 5.675 | 3.023 | 0 | 0.016 | 0.018 |
| slvk1-sp | 0.653 | 0.993 | 26.534 | 20.07 | 6.443 | 0.172 | 0.016 | 5.732 | 2.176 | 0.189 | 0.032 | 0 |
| slvk1-sp | 0.462 | 0.949 | 28.21 | 18.392 | 6.976 | 0.203 | 0.016 | 4.946 | 2.039 | 0.008 | 0.109 | 0.026 |
| slvk1-sp | 0.084 | 1.369 | 25.192 | 20.952 | 6.151 | 0.092 | 0.015 | 4.985 | 2.678 | 0 | 0.02 | 0.07 |
| slvk1-sp | 0.413 | 0.552 | 25.696 | 24.371 | 4.901 | 0.182 | 0 | 3.869 | 1.407 | 0 | 0.077 | 0.026 |
| slvk1-sp | 0.355 | 0.439 | 25.989 | 20.282 | 5.635 | 1.213 | 0.067 | 5.437 | 1.782 | 0.114 | 0.101 | 0.114 |
| slvk1-sp | 0.169 | 0.728 | 27.333 | 14.94 | 8.783 | 0.281 | 0 | 7.397 | 1.515 | 0.051 | 0.045 | 0.009 |
| slvk1-sp | 0.129 | 0.69 | 23.278 | 20.675 | 6.723 | 1.247 | 0.09 | 5.194 | 2.651 | 0.269 | 0.02 | 0.061 |
| slvk1-sp | 0.407 | 0.443 | 26.465 | 20.443 | 5.025 | 0.844 | 0.102 | 4.934 | 2.118 | 0.241 | 0.113 | 0 |
| slvk1-sp | 0.707 | 1.15 | 25.727 | 20.243 | 5.616 | 0.088 | 0 | 4.197 | 1.963 | 0 | 0.081 | 0.009 |
| slvk1-sp | 0.457 | 0.821 | 32.315 | 12.606 | 5.183 | 0.66 | 0.018 | 4.35 | 0.928 | 0.114 | 0 | 0 |
| slvk1-sp | 0.656 | 1.196 | 31.386 | 12.889 | 5.014 | 0.186 | 0.015 | 2.701 | 1.159 | 0.016 | 0.008 | 0 |
| slvk2-sp | 0.214 | 0.392 | 18.229 | 27.279 | 8.545 | 0.24 | 0.017 | 12.31 | 2.64 | 0.167 | 0.171 | 0.035 |
| slvk2-sp | 0.63 | 1.385 | 25.386 | 22.622 | 5.82 | 1.471 | 0.477 | 7.463 | 3.217 | 0.47 | 0.061 | 0.061 |
| slvk2-sp | 0.315 | 3.207 | 20.014 | 24.421 | 4.461 | 4.208 | 1.452 | 5.154 | 2.801 | 0.859 | 0.138 | 0.018 |
| slvk2-sp | 0.394 | 2.613 | 22.443 | 23.385 | 6.477 | 0.398 | 0 | 7.058 | 3.336 | 0.063 | 0.089 | 0.035 |
| slvk2-sp | 0.263 | 0.747 | 26.682 | 19.845 | 6.696 | 1.204 | 0.28 | 7.926 | 2.595 | 0.277 | 0.105 | 0 |
| slvk2-sp | 0.286 | 0.837 | 24.657 | 23.695 | 6.046 | 1.721 | 0.035 | 6.742 | 2.433 | 0.055 | 0.102 | 0.018 |
| slvk2-sp | 0.498 | 0.396 | 28.165 | 19.309 | 7.066 | 0.176 | 0.022 | 7.717 | 1.879 | 0.071 | 0.069 | 0 |
| slvk2-sp | 0.58 | 2.732 | 29.76 | 15.959 | 7.467 | 0.094 | 0.002 | 6.001 | 2.441 | 0.103 | 0.101 | 0.053 |
| slvk2-sp | 0.341 | 3.014 | 23.389 | 19.049 | 8.06 | 2.477 | 0.031 | 4.982 | 3.061 | 0.246 | 0.077 | 0.07 |
| slvk2-sp | 0.307 | 0.574 | 23.91 | 16.781 | 7.335 | 8.57 | 0.403 | 3.622 | 2.388 | 0.666 | 0.081 | 0.07 |
| slvk2-sp | 0.444 | 0.739 | 30.173 | 20.397 | 6.026 | 0.204 | 0 | 4.284 | 2.15 | 0.142 | 0 | 0.009 |
| slvk2-sp | 0.169 | 2.328 | 18.311 | 26.294 | 5.111 | 2.89 | 0.689 | 4.587 | 3.1 | 0.471 | 0.073 | 0.088 |
| slvk2-sp | 0.616 | 0.787 | 24.875 | 23.296 | 6.42 | 0.156 | 0.007 | 5.414 | 2.302 | 0.012 | 0.122 | 0.097 |
| slvk2-sp | 0.934 | 1.358 | 29.635 | 16.981 | 4.542 | 0.347 | 0.012 | 3.825 | 2.016 | 4.247 | 0.136 | 0 |
| slvk2-sp | 0.531 | 1.552 | 23.094 | 22.184 | 5.923 | 0.376 | 0.1 | 6.956 | 2.658 | 0.3 | 0.065 | 0.061 |
| slvk1995-1 | 0.444 | 1.659 | 23.951 | 23.685 | 7.497 | 0.619 | 0.028 | 4.489 | 2.009 | 0.036 | 0.132 | 0 |
| slvk1995-1 | 0.564 | 1.281 | 23.118 | 21.357 | 10.055 | 0.279 | 0 | 6.554 | 1.773 | 0.036 | 0.221 | 0 |
| slvk1995-1 | 0.192 | 3.435 | 19.469 | 21.071 | 4.751 | 0.396 | 0.032 | 3.366 | 1.169 | 0.015 | 0.074 | 0 |
| slvk1995-1 | 0.233 | 6.445 | 18.23 | 18.002 | 10.206 | 0.214 | 0.165 | 3.906 | 1.459 | 0 | 0.027 | 0 |
| slvk1995-1 | 0.367 | 6.248 | 17.371 | 21.269 | 10.217 | 0.167 | 0.009 | 5.321 | 1.221 | 0.015 | 0.117 | 0 |
| slvk1995-1 | 0.296 | 2.932 | 19.298 | 22.95 | 10.259 | 0.244 | 0.005 | 5.534 | 1.176 | 0.066 | 0.14 | 0.072 |
| slvk1995-1 | 0.454 | 1.052 | 19.902 | 20.39 | 7.013 | 2.604 | 0.579 | 4.592 | 2.097 | 1.067 | 0.236 | 0.12 |
| slvk1995-1 | 0.451 | 1.532 | 20.029 | 23.011 | 9.251 | 0.631 | 0.043 | 4.384 | 1.694 | 0.088 | 0.144 | 0.04 |
| slvk1995-1 | 0.556 | 1.334 | 21.84 | 21.829 | 10.344 | 0.657 | 0.011 | 6.192 | 1.462 | 0.026 | 0.113 | 0 |
| slvk1995-1 | 0.498 | 2.337 | 22.004 | 22.641 | 9.952 | 0.581 | 0.015 | 5.423 | 1.577 | 0.066 | 0.116 | 0.032 |
| slvk1995-1 | 0.353 | 1.298 | 20.955 | 21.855 | 9.964 | 0.357 | 0.007 | 5.491 | 1.379 | 0 | 0.159 | 0 |
| slvk1995-1 | 1.274 | 1.396 | 21.252 | 19.778 | 12.185 | 3.322 | 0.005 | 4.791 | 1.41 | 0.018 | 0.154 | 0.031 |
| slvk1995-1 | 0.274 | 2.406 | 20.215 | 24.268 | 10.729 | 0.665 | 0.013 | 6.111 | 1.76 | 0.102 | 0.14 | 0.016 |
| slvk1995-1 | 0.536 | 1.221 | 20.392 | 23.583 | 8.551 | 1.889 | 0.032 | 4.419 | 1.627 | 0.168 | 0.113 | 0 |
| slvk1995-1 | 0.262 | 2.453 | 17.232 | 28.438 | 9.177 | 0.579 | 0.017 | 5.254 | 1.902 | 0.018 | 0.086 | 0 |
| slvk1995-2 | 0.897 | 2.359 | 22.342 | 19.174 | 9.579 | 0.242 | 0.013 | 4.105 | 1.253 | 0.102 | 0.097 | 0 |
| slvk1995-2 | 0.459 | 1.788 | 22.093 | 23.108 | 9.191 | 0.189 | 0.017 | 5.433 | 1.477 | 0.018 | 0.062 | 0.008 |
| slvk1995-2 | 0.669 | 3.272 | 20.694 | 22.032 | 9.52 | 1.669 | 0.155 | 5.014 | 1.391 | 0.284 | 0.171 | 0.12 |
| slvk1995-2 | 0.795 | 1.635 | 22.557 | 22.176 | 8.768 | 0.333 | 0.03 | 5.599 | 1.426 | 0.036 | 0.124 | 0.024 |
| slvk1995-2 | 0.492 | 1.029 | 22.704 | 25.462 | 5.871 | 0.325 | 0.006 | 4.195 | 0.953 | 0 | 0.089 | 0.072 |
| slvk1995-2 | 0.503 | 0.67 | 21.227 | 29.47 | 5.558 | 0.374 | 0.014 | 3.483 | 1.49 | 0.047 | 0.136 | 0.04 |
| slvk1995-2 | 0.643 | 2.382 | 23.226 | 21.263 | 9.217 | 0.347 | 0.005 | 4.733 | 1.395 | 0.047 | 0.163 | 0.008 |
| slvk1995-2 | 0.188 | 0.689 | 18.159 | 24.409 | 19.069 | 0.455 | 0.019 | 9.274 | 2.04 | 0.04 | 0.199 | 0 |
| slvk1995-2 | 0.75 | 1.958 | 26.861 | 20.638 | 5.68 | 0.187 | 0.01 | 2.869 | 1.627 | 0 | 0.093 | 0 |
| slvk1995-2 | 1.079 | 1.527 | 26.755 | 16.292 | 8.072 | 0.098 | 0.005 | 5.077 | 0.907 | 0.073 | 0.135 | 0.024 |
| slvk1995-2 | 0.369 | 1.128 | 21.096 | 21.856 | 8.707 | 0.649 | 0.017 | 3.975 | 1.233 | 0.044 | 0.054 | 0.032 |
| slvk1995-2 | 0.504 | 1.839 | 21.838 | 19.08 | 7.109 | 0.376 | 0.002 | 4.015 | 1.046 | 0.058 | 0.167 | 0 |
| slvk1995-2 | 0.323 | 1.337 | 18.647 | 23.802 | 12.192 | 0.414 | 0 | 6.559 | 1.569 | 0 | 0.148 | 0 |
| slvk1995-2 | 0.384 | 1.11 | 21.178 | 22.467 | 9.181 | 0.372 | 0.026 | 4.937 | 1.417 | 0.029 | 0.136 | 0.032 |
| slvk1995-2 | 0.443 | 1.916 | 22.429 | 22.553 | 8.602 | 1.534 | 0.128 | 5.519 | 1.286 | 0.149 | 0.116 | 0 |

**Table 1S. Data for the multivariate discriminant analysis.** The full set of data included 95x12 values for the Stradivarius violin, 75x12 for the early Guarneri and 30x12 for the rest, with the exception of the German maple which had only 15x12 data points. The data set from the pellets from each musical instrument was analyzed in its entirety as one group, while the commercial woods were analyzed as sets of 15. Abbreviations: Guarn: the early Guarneri violin; Strad: Stradivari; StrCello: Stradivari cello; Gand: Gand-Bernardel violin. The following commercial wood ashes were analyzed: Bosn2M1 and Bosn2M2 are 2 groups of 15 sites each from the Bosnian tree no. 2; similarly, Bosn3M1, Bosn3M2 are 2 groups of 15 from the Bosnian tree no. 3 tree and so on up to Bosn6M1 and Bosn6M2; ChinaM1 and ChinaM2 are 2 groups of 15 sites from the ash pellet of one Chinese maple; GerMaple has only15 sites from the pellet of the German maple; slven1-ht and slven1-sp are groups of 15 sites from the heartwood and sapwood of a Slovenian maple, and slven2-ht and slven2-sp are 2 groups of 15 from the same board but a deeper layer of wood; slvk1-ht and slvk1-sp are 15 sites each from the heartwood and sapwood of a Slovakian maple, and slvk2-ht and slvk2-sp represent sites from the same board but a deeper layer; slvk1995-1 and slvk1995-2 designate 2 groups of 15 sites from a different Slovakian maple board obtained in 1995.
